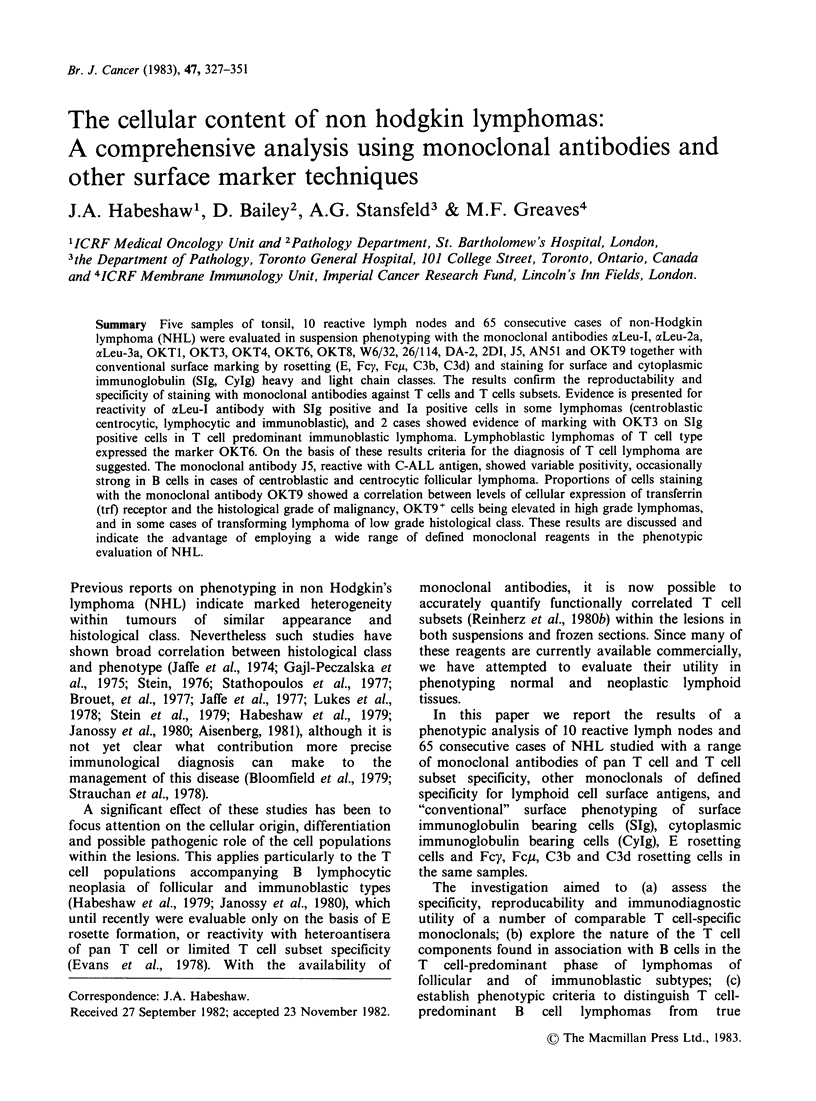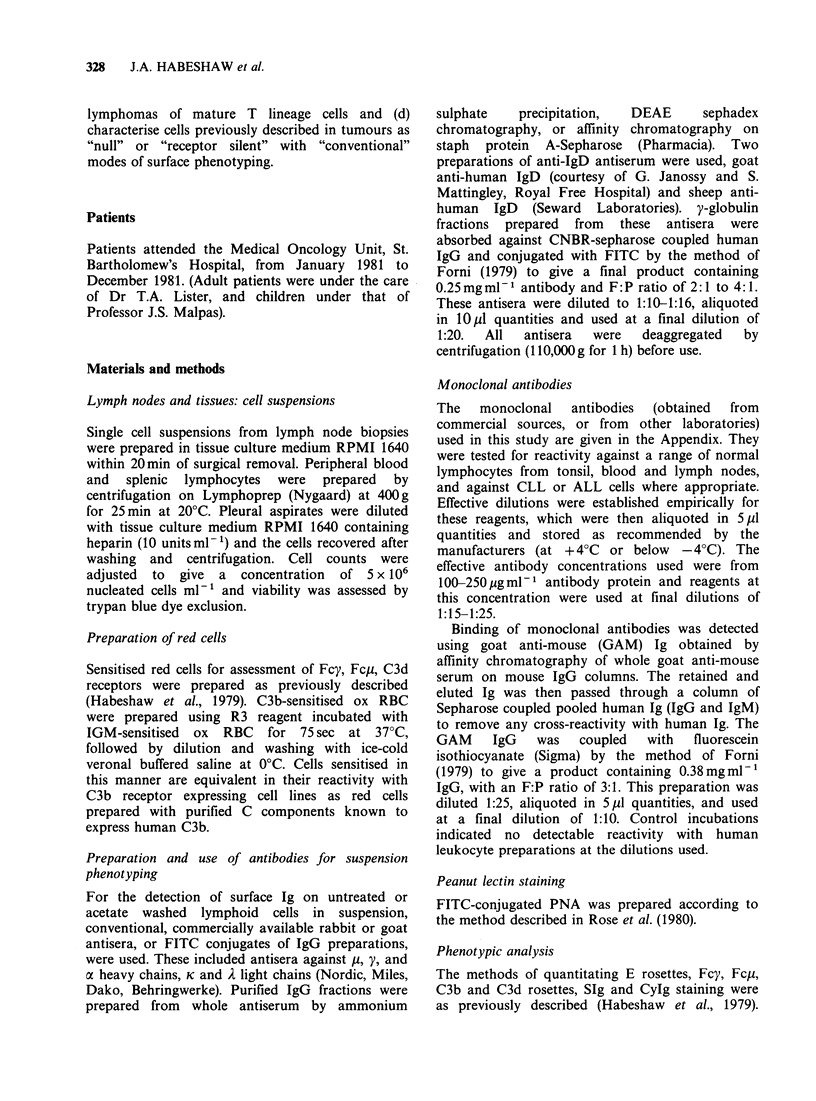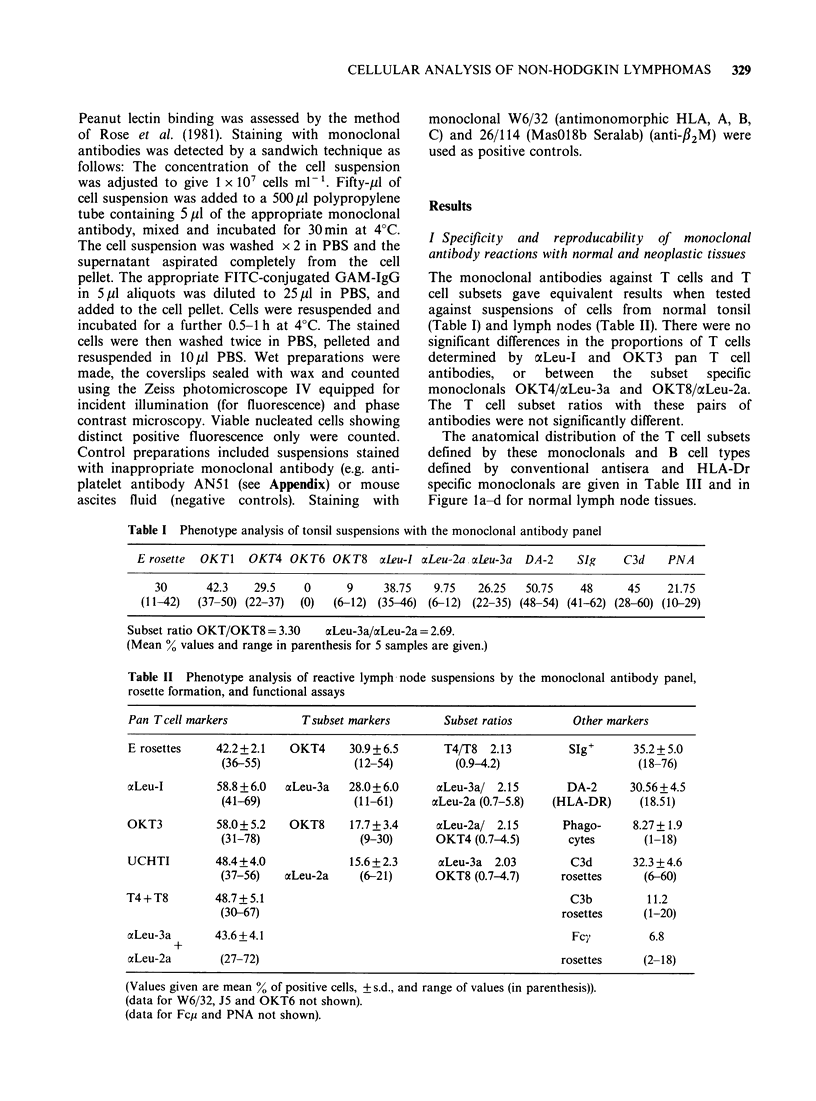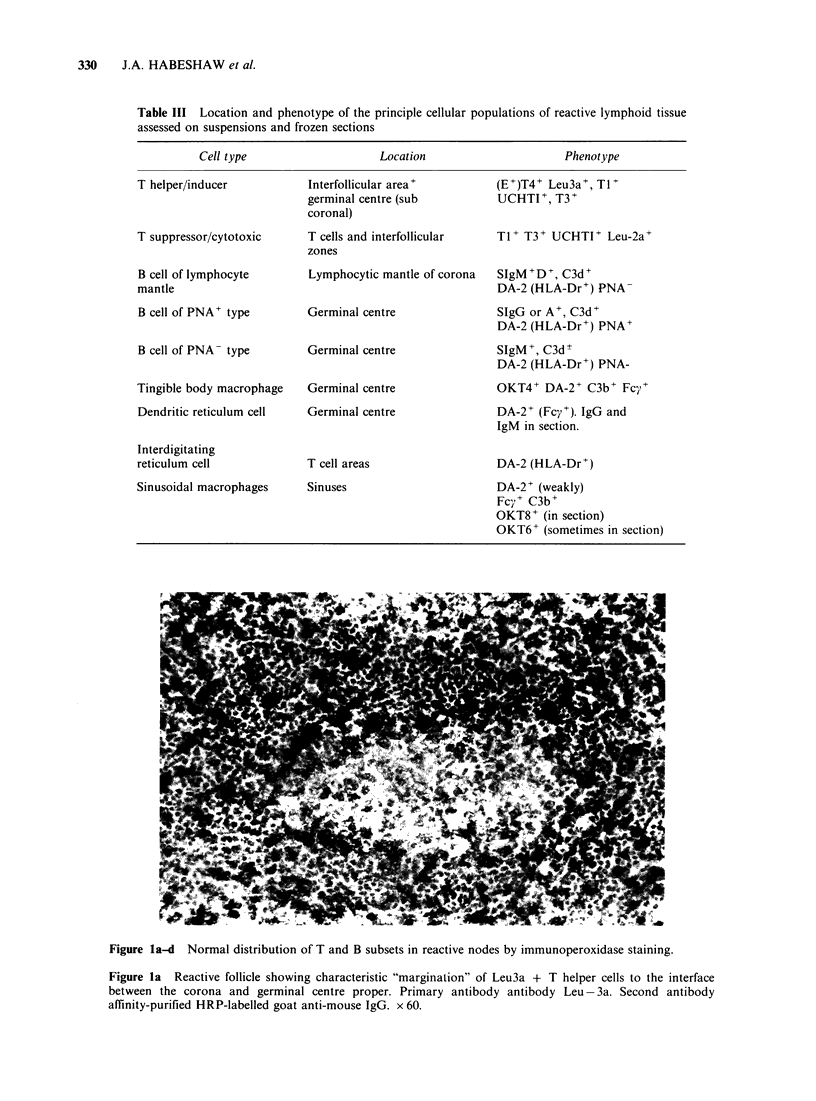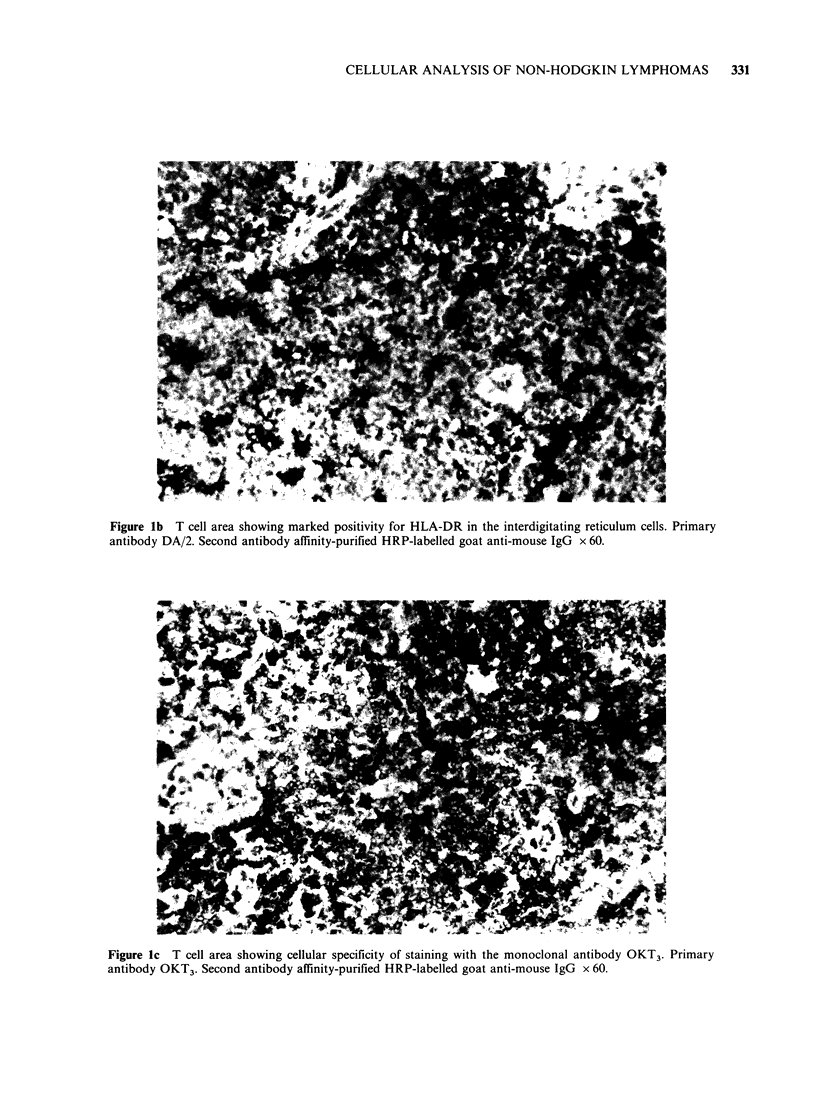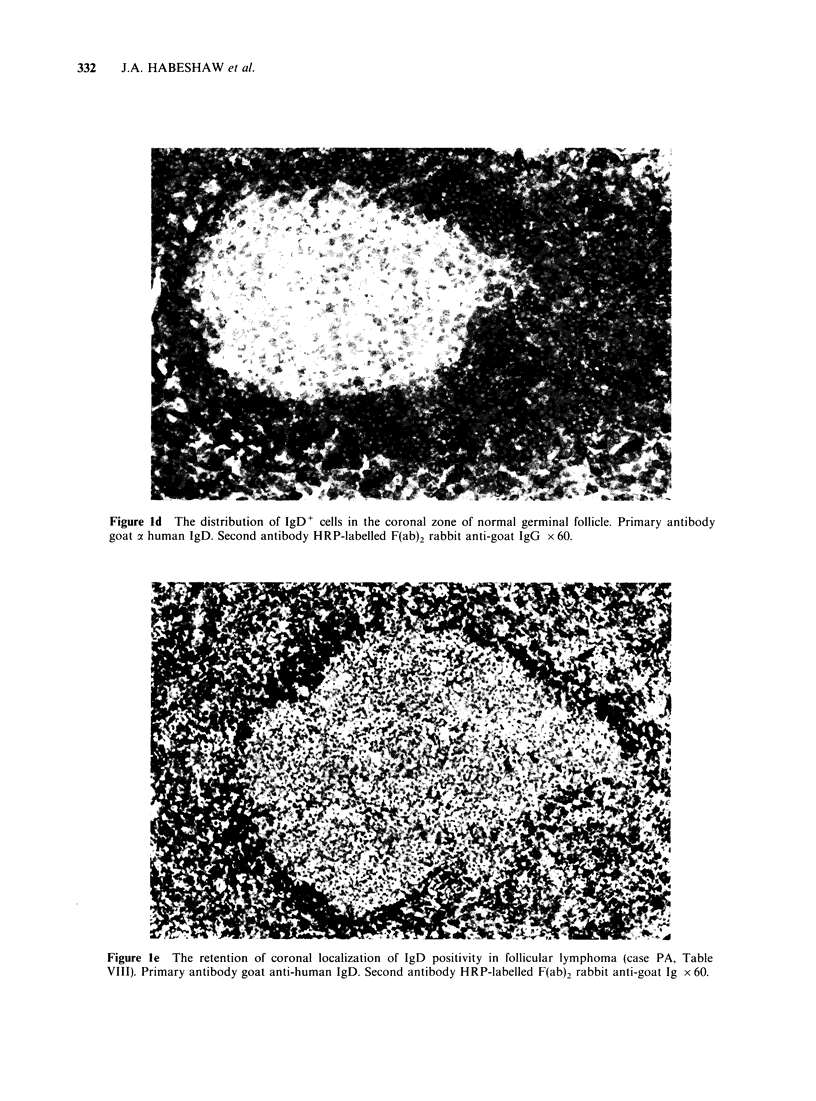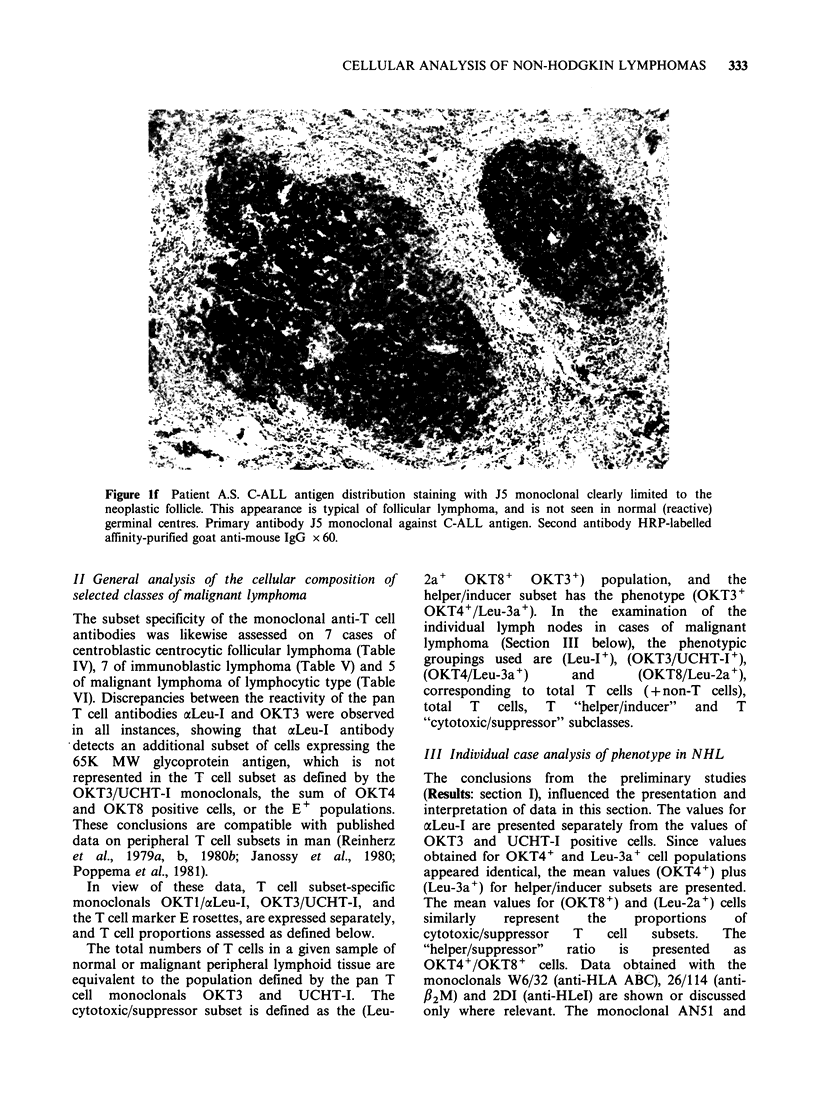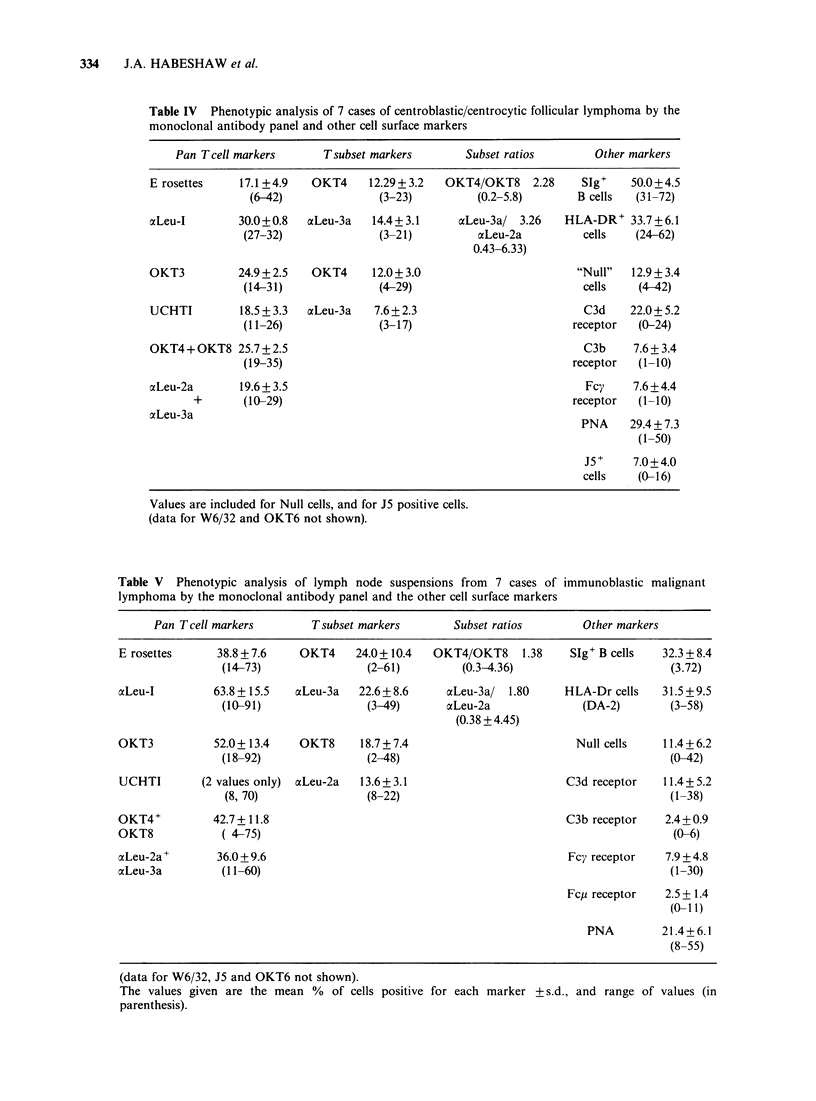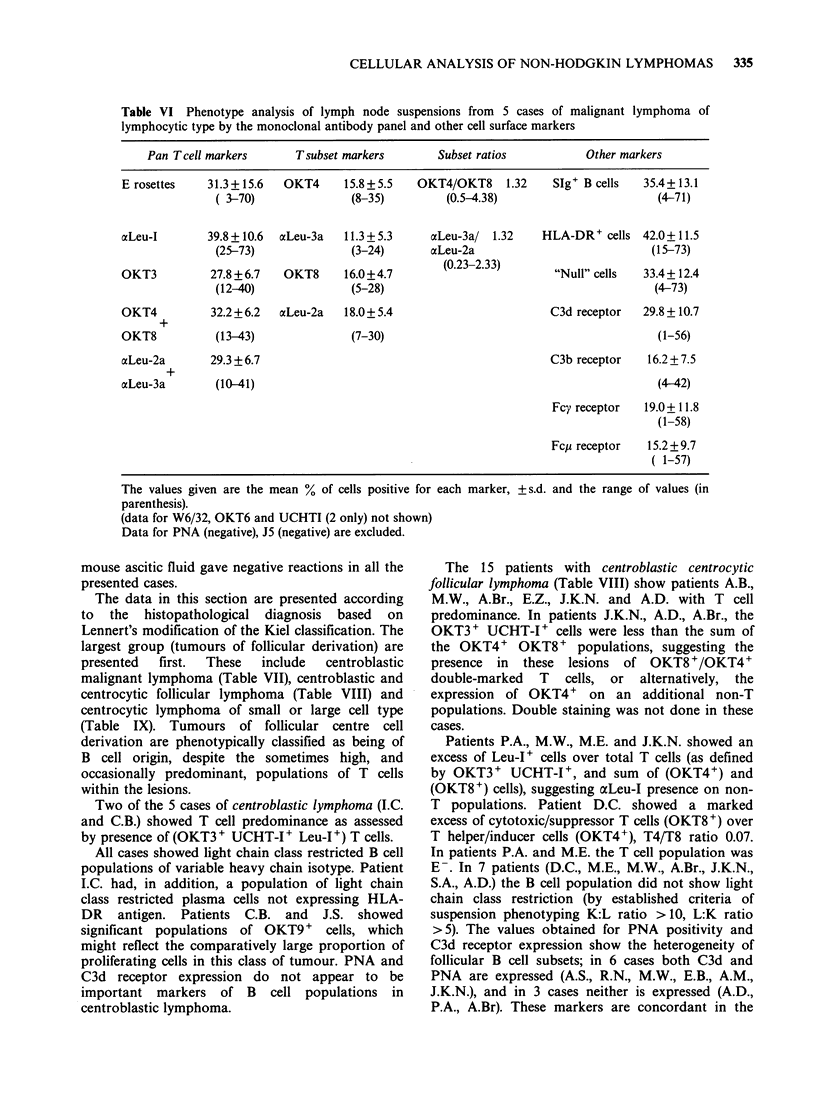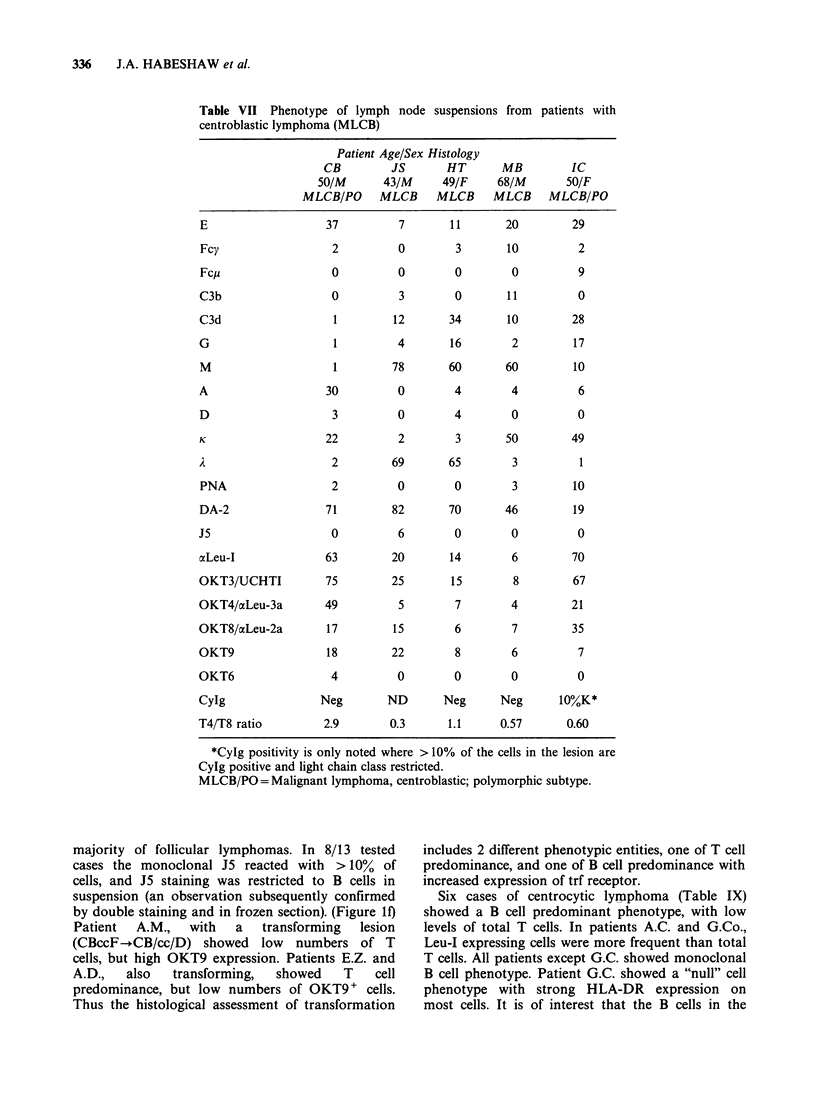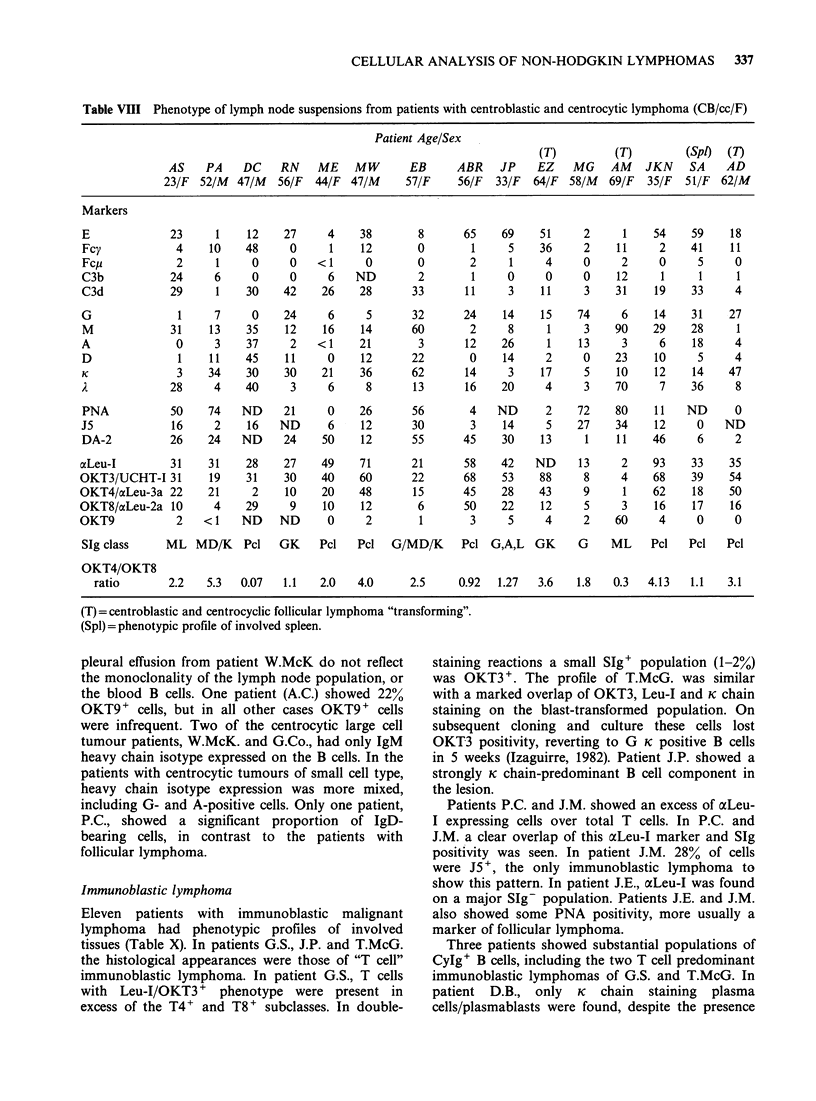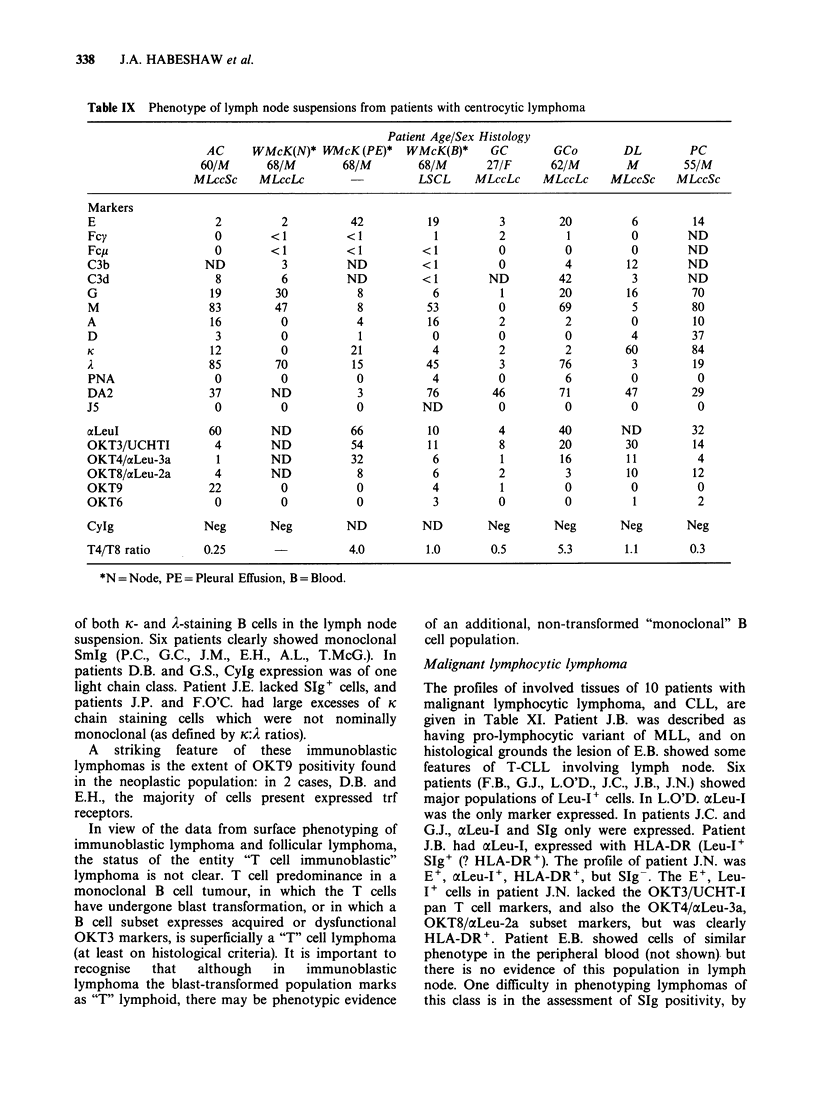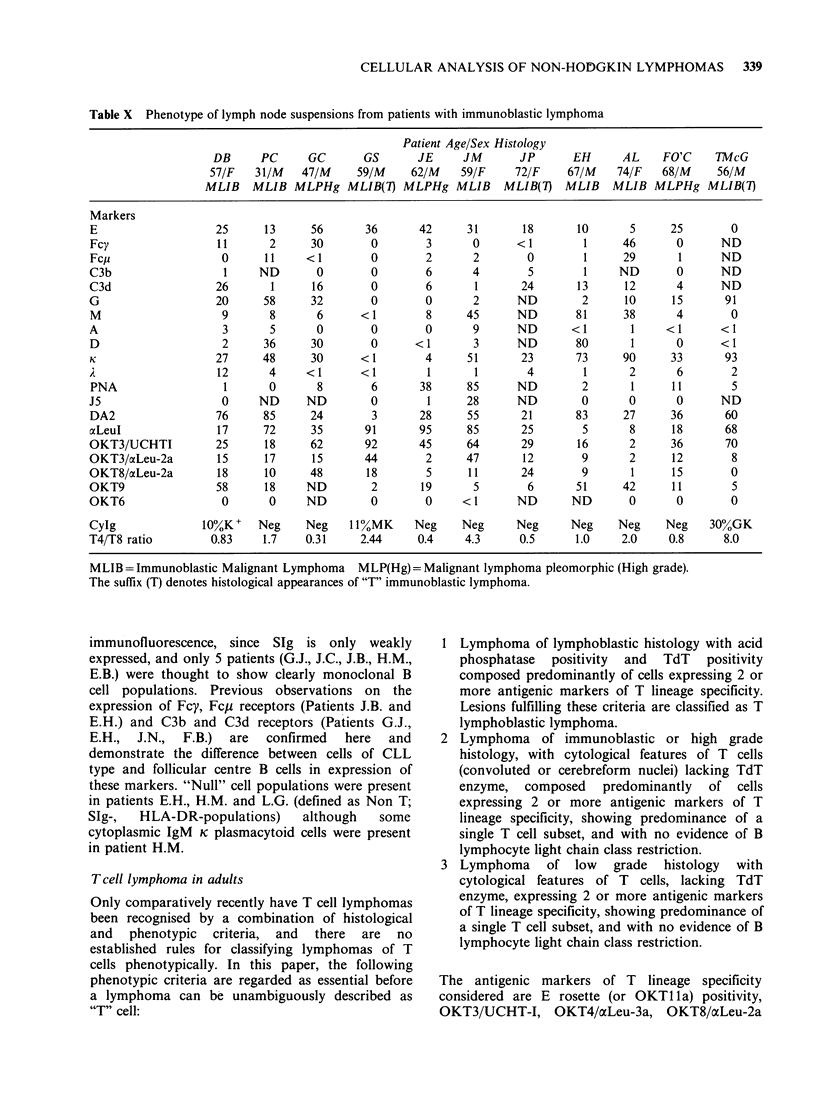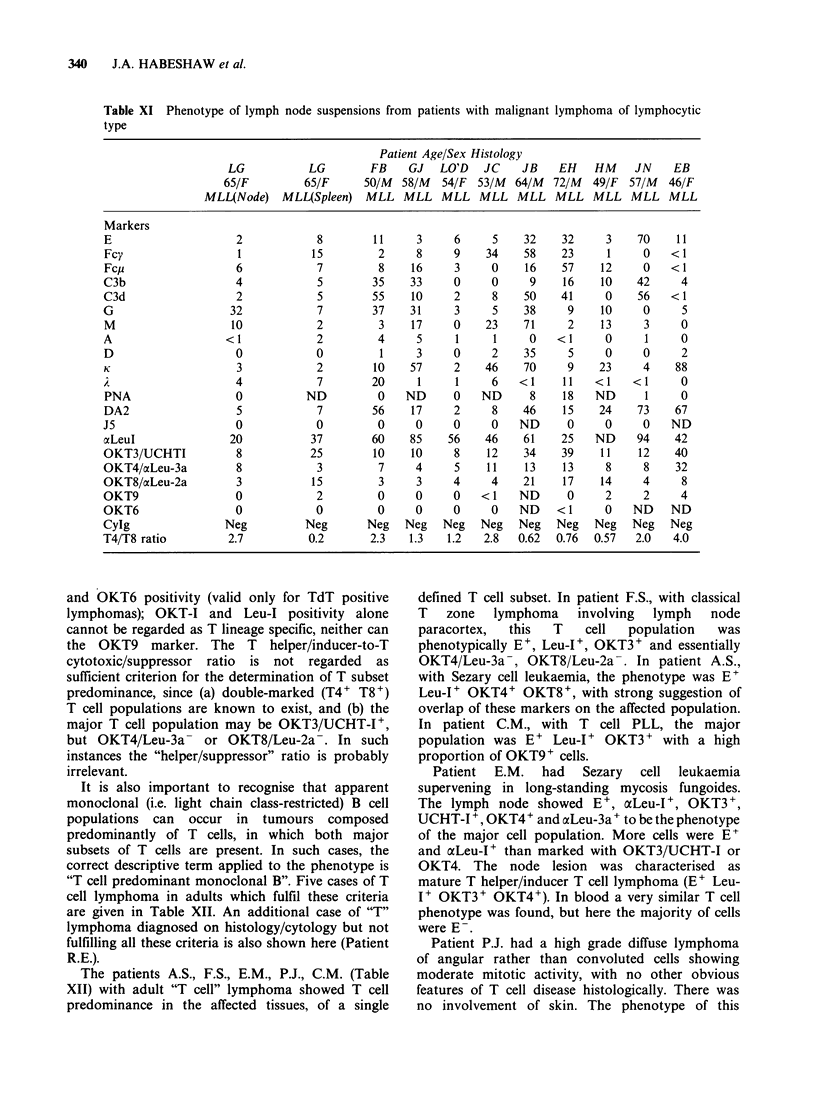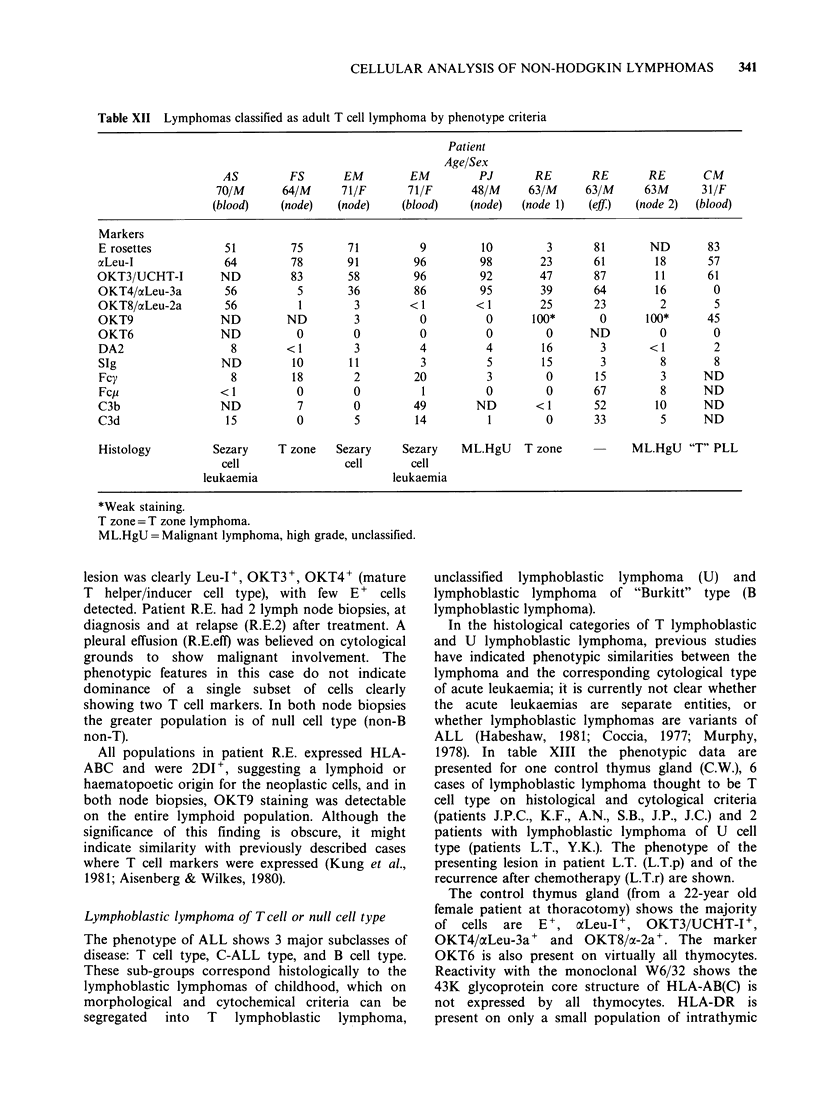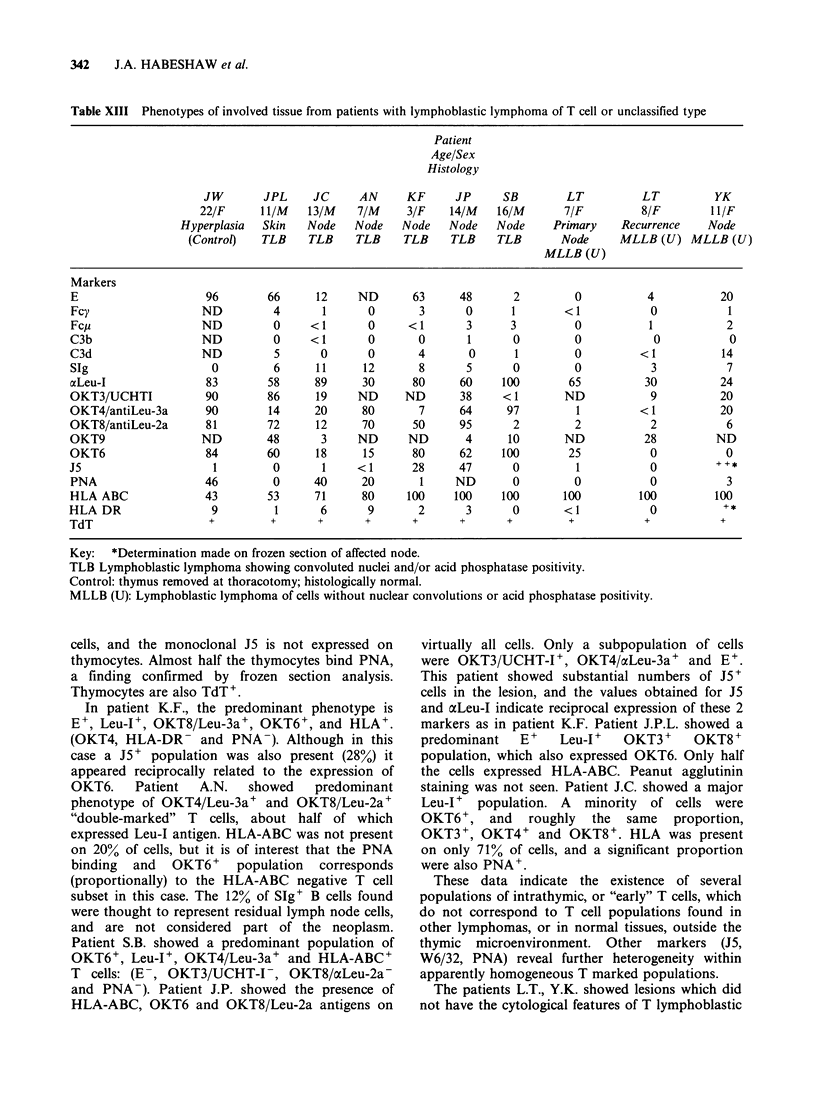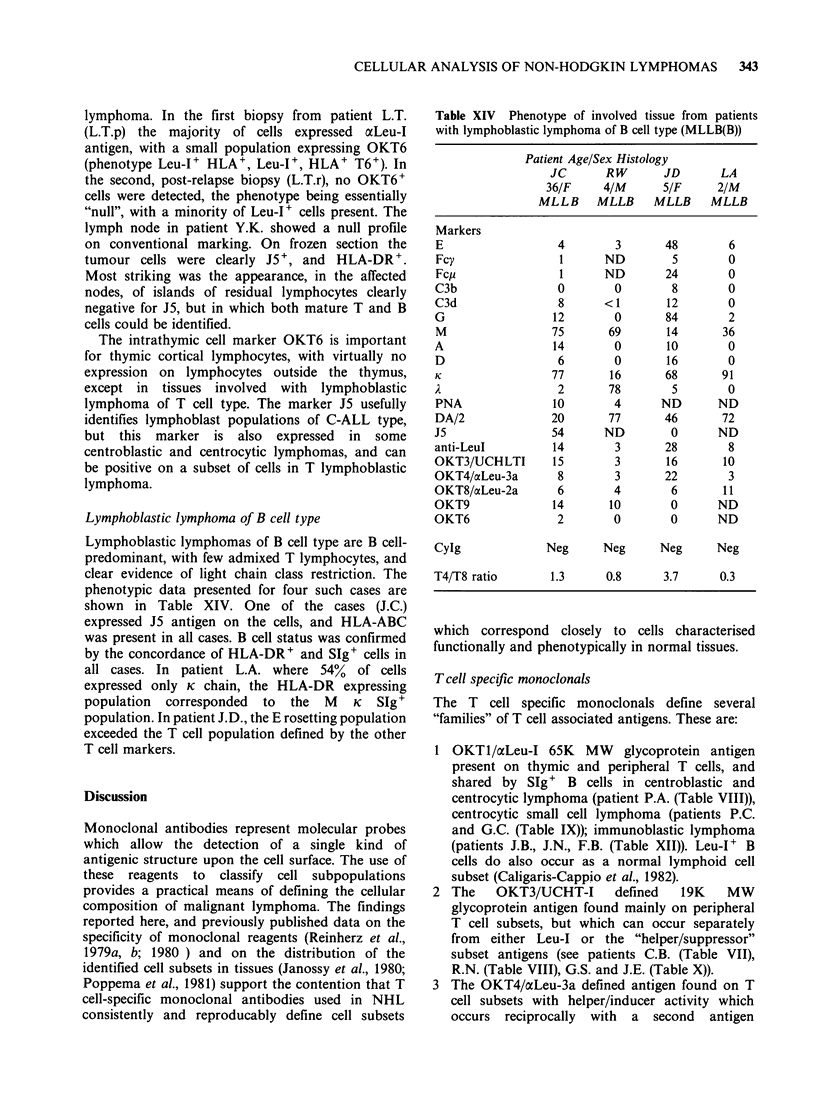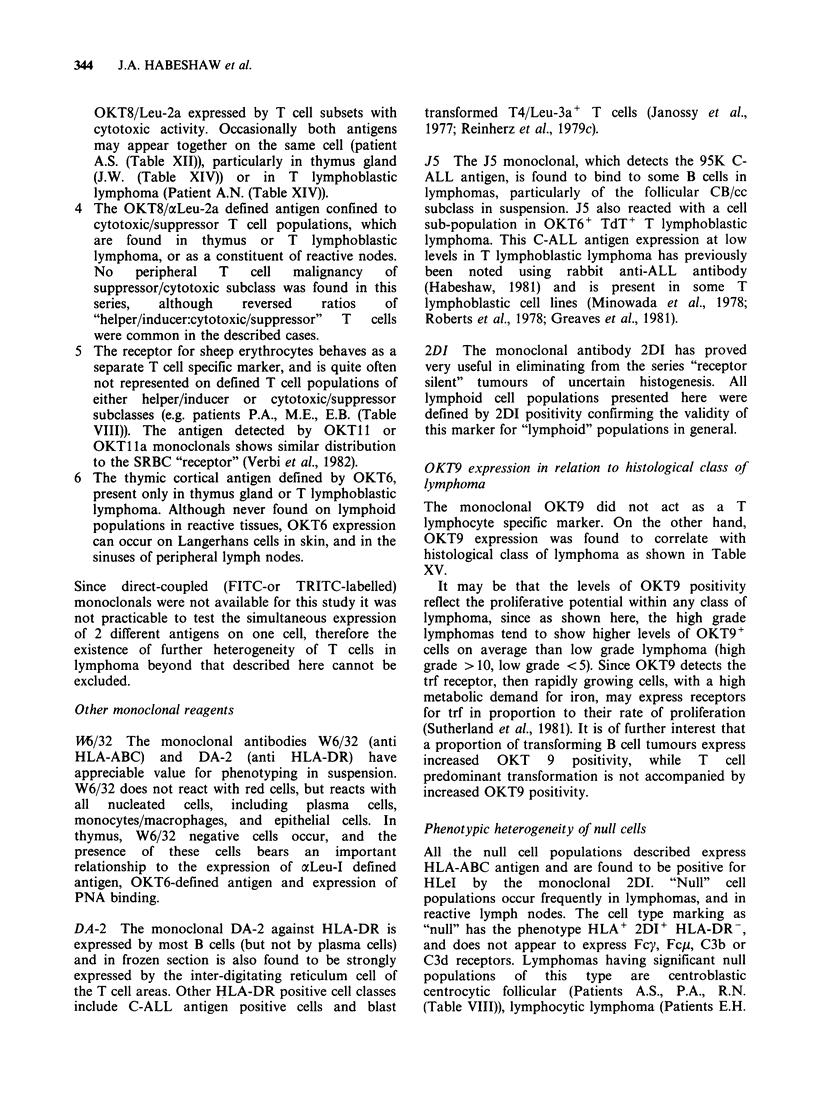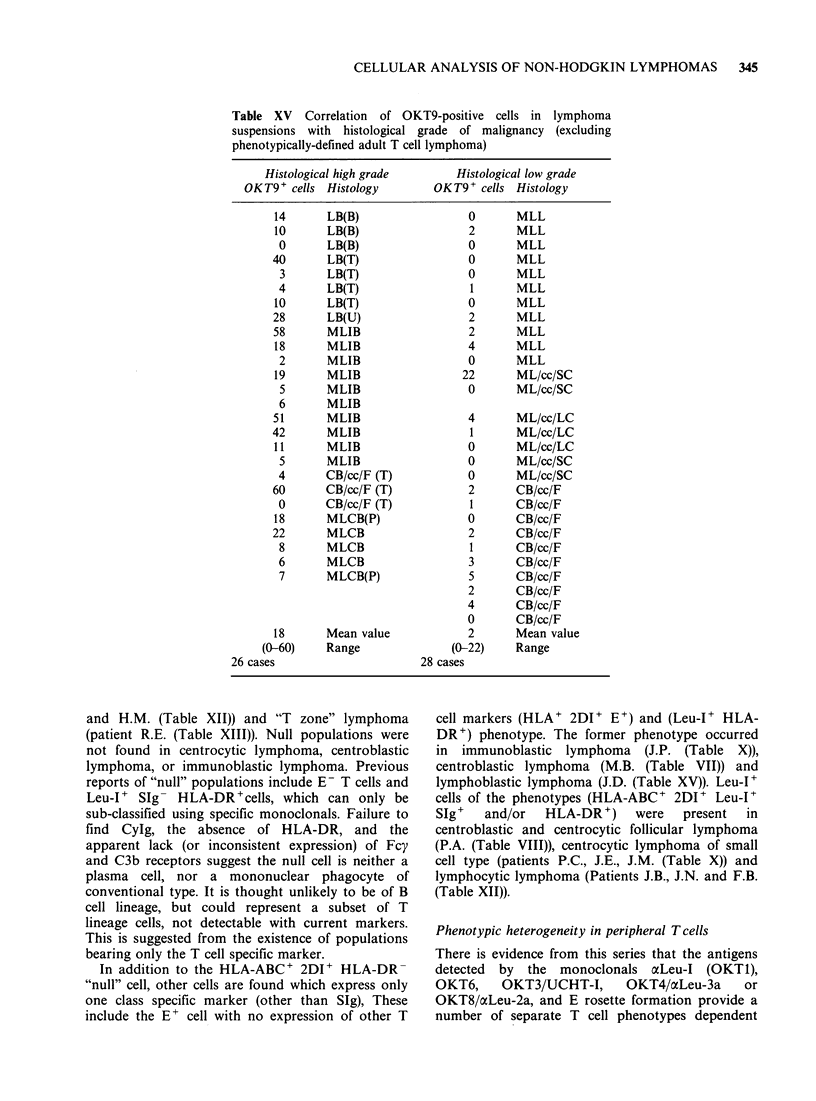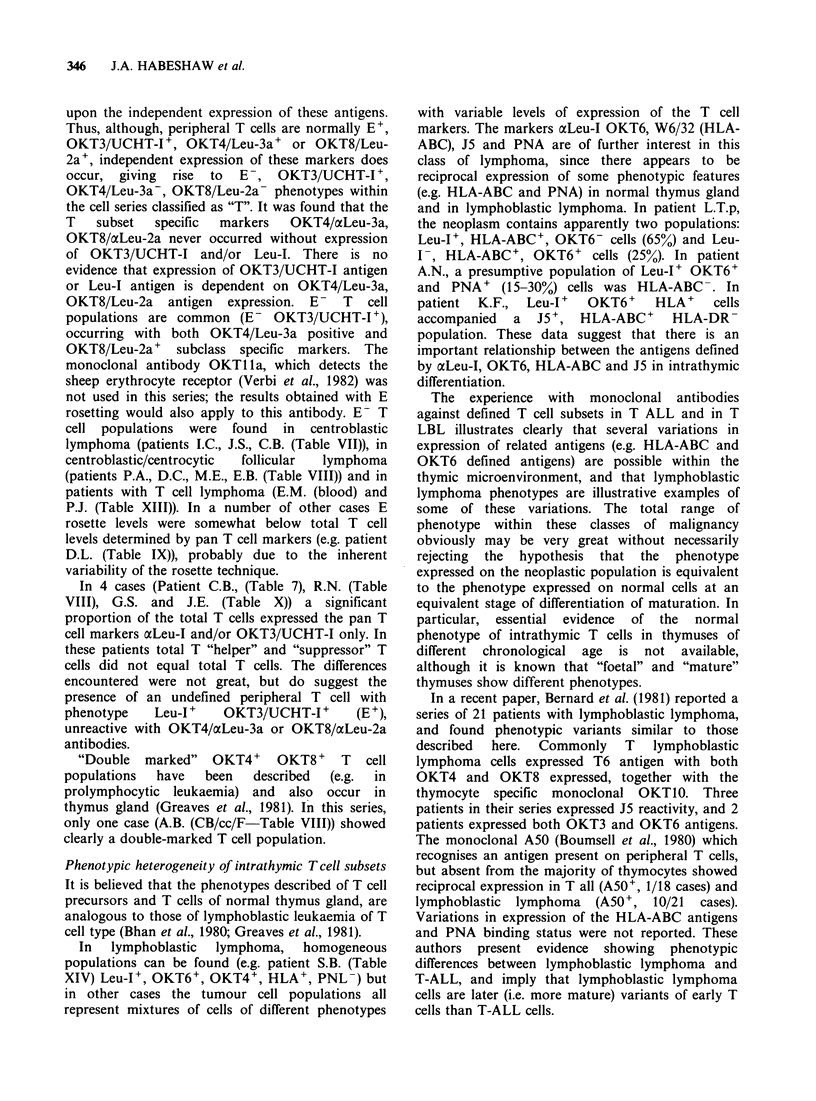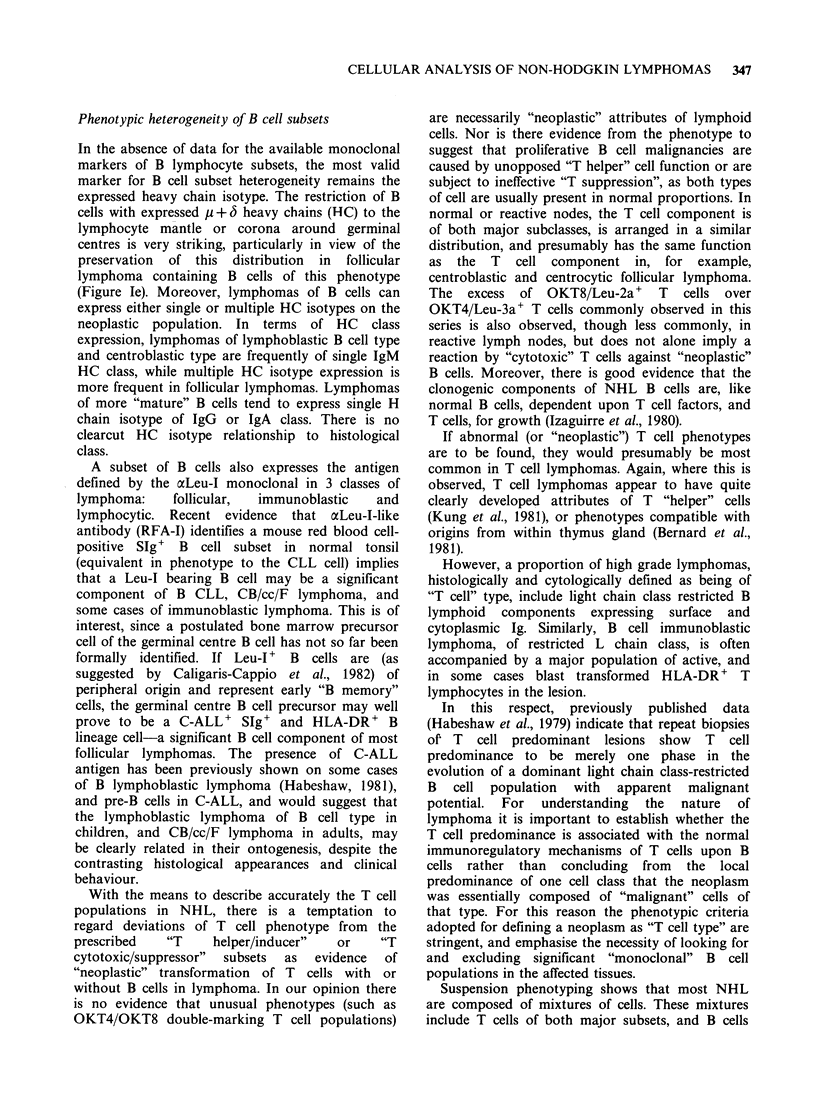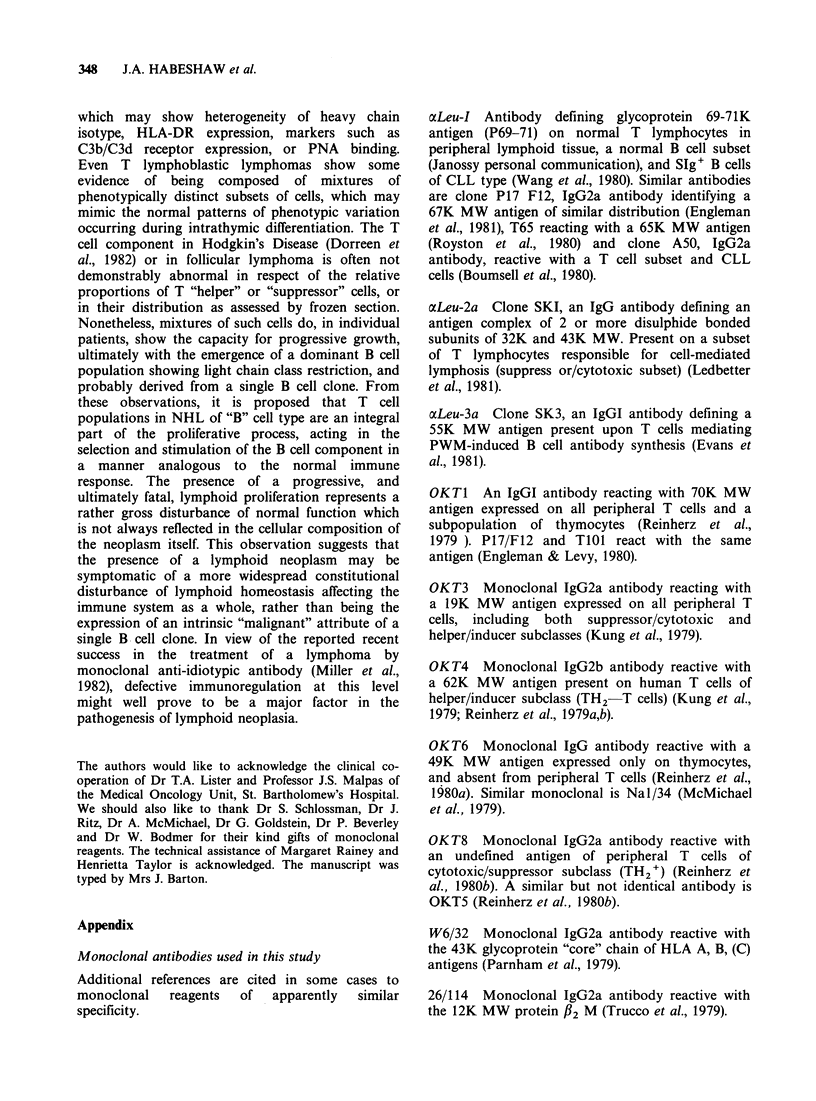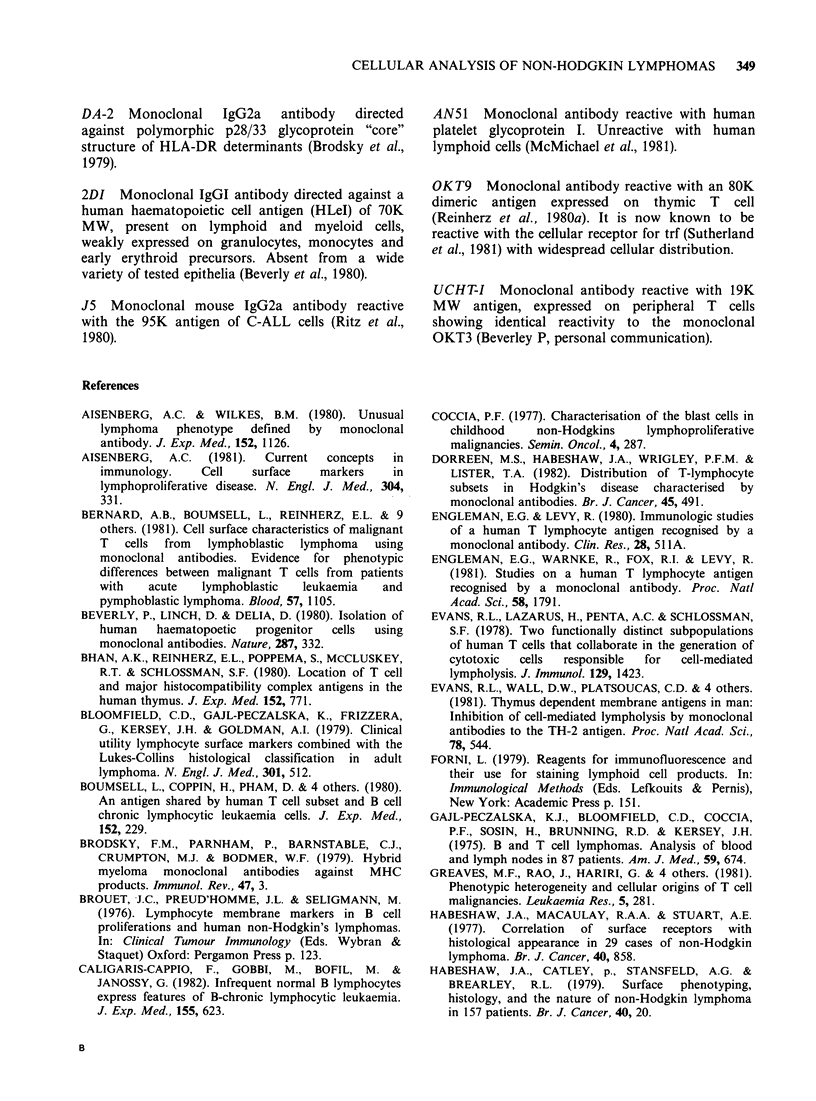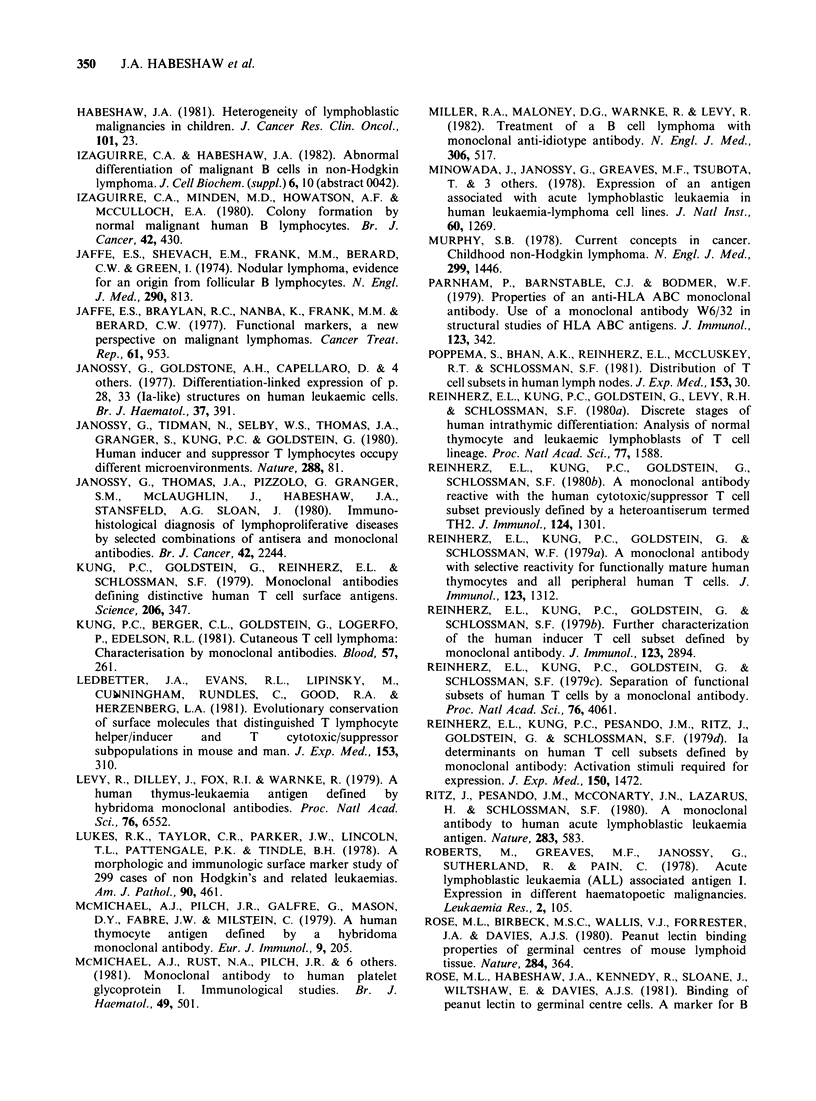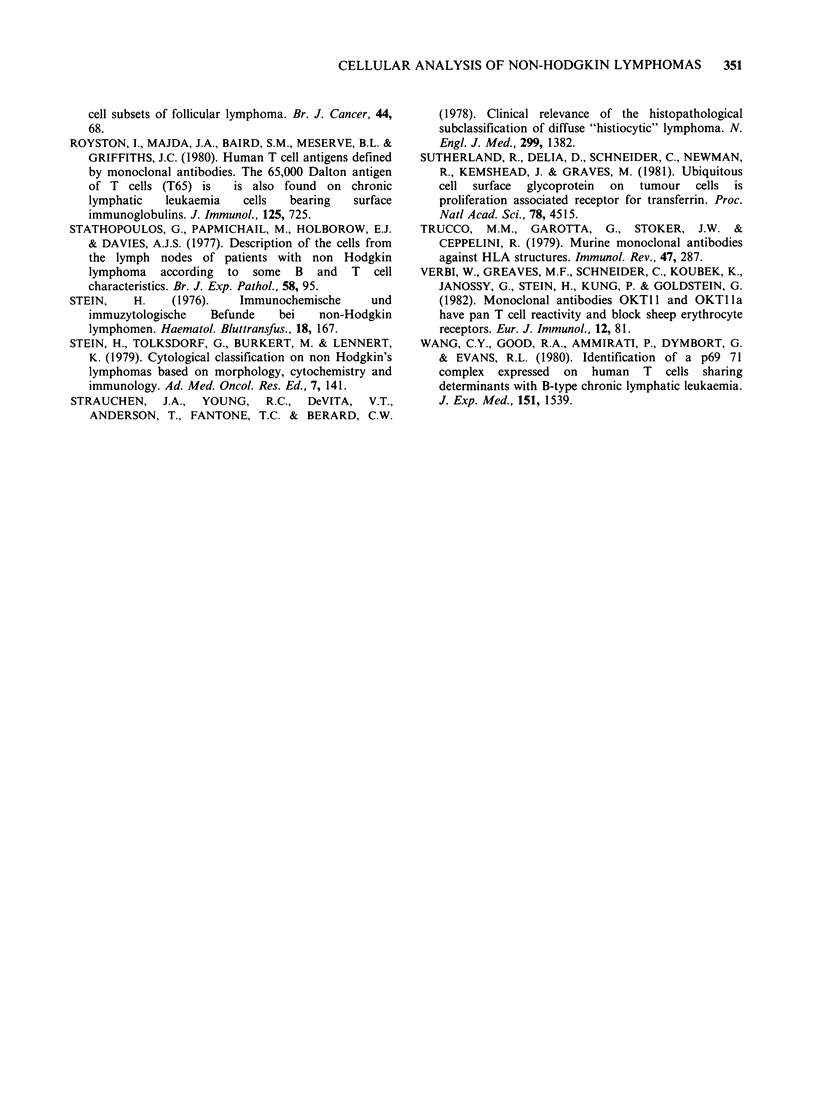# The cellular content of non Hodgkin lymphomas: a comprehensive analysis using monoclonal antibodies and other surface marker techniques.

**DOI:** 10.1038/bjc.1983.52

**Published:** 1983-03

**Authors:** J. A. Habeshaw, D. Bailey, A. G. Stansfeld, M. F. Greaves

## Abstract

**Images:**


*Br. J. Cancer* (1983), **47**, 327–351

### The cellular content of non hodgkin lymphomas: A comprehensive analysis using monoclonal antibodies and other surface marker techniques

J.A. Habeshaw[1], D. Bailey[2], A.G. Stansfeld[3] & M.F. Greaves[4]

[1]*ICRF Medical Oncology Unit and* [2]*Pathology Department, St. Bartholomew's Hospital, London,* [3]*the Department of Pathology, Toronto General Hospital, 101 College Street, Toronto, Ontario, Canada and* [4]*ICRF Membrane Immunology Unit, Imperial Cancer Research Fund, Lincoln's Inn Fields, London.*

**Summary** Five samples of tonsil, 10 reactive lymph nodes and 65 consecutive cases of non-Hodgkin lymphoma (NHL) were evaluated in suspension phenotyping with the monoclonal antibodies αLeu-I, αLeu-2a, αLeu-3a, OKT1, OKT3, OKT4, OKT6, OKT8, W6/32, 26/114, DA-2, 2DI, J5, AN51 and OKT9 together with conventional surface marking by rosetting (E, Fcγ, Fcμ, C3b, C3d) and staining for surface and cytoplasmic immunoglobulin (SIg, CyIg) heavy and light chain classes. The results confirm the reproductability and specificity of staining with monoclonal antibodies against T cells and T cells subsets. Evidence is presented for reactivity of αLeu-I antibody with SIg positive and Ia positive cells in some lymphomas (centroblastic centrocytic, lymphocytic and immunoblastic), and 2 cases showed evidence of marking with OKT3 on SIg positive cells in T cell predominant immunoblastic lymphoma. Lymphoblastic lymphomas of T cell type expressed the marker OKT6. On the basis of these results criteria for the diagnosis of T cell lymphoma are suggested. The monoclonal antibody J5, reactive with C-ALL antigen, showed variable positivity, occasionally strong in B cells in cases of centroblastic and centrocytic follicular lymphoma. Proportions of cells staining with the monoclonal antibody OKT9 showed a correlation between levels of cellular expression of transferrin (trf) receptor and the histological grade of malignancy, $OKT9^+$ cells being elevated in high grade lymphomas, and in some cases of transforming lymphoma of low grade histological class. These results are discussed and indicate the advantage of employing a wide range of defined monoclonal reagents in the phenotypic evaluation of NHL.

Previous reports on phenotyping in non Hodgkin's lymphoma (NHL) indicate marked heterogeneity within tumours of similar appearance and histological class. Nevertheless such studies have shown broad correlation between histological class and phenotype (Jaffe *et al.*, 1974; Gajl-Peczalska *et al.*, 1975; Stein, 1976; Stathopoulos *et al.*, 1977; Brouet, *et al.*, 1977; Jaffe *et al.*, 1977; Lukes *et al.*, 1978; Stein *et al.*, 1979; Habeshaw *et al.*, 1979; Janossy *et al.*, 1980; Aisenberg, 1981), although it is not yet clear what contribution more precise immunological diagnosis can make to the management of this disease (Bloomfield *et al.*, 1979; Strauchan *et al.*, 1978).

A significant effect of these studies has been to focus attention on the cellular origin, differentiation and possible pathogenic role of the cell populations within the lesions. This applies particularly to the T cell populations accompanying B lymphocytic neoplasia of follicular and immunoblastic types (Habeshaw *et al.*, 1979; Janossy *et al.*, 1980), which until recently were evaluable only on the basis of E rosette formation, or reactivity with heteroantisera of pan T cell or limited T cell subset specificity (Evans *et al.*, 1978). With the availability of monoclonal antibodies, it is now possible to accurately quantify functionally correlated T cell subsets (Reinherz *et al.*, 1980*b*) within the lesions in both suspensions and frozen sections. Since many of these reagents are currently available commercially, we have attempted to evaluate their utility in phenotyping normal and neoplastic lymphoid tissues.

In this paper we report the results of a phenotypic analysis of 10 reactive lymph nodes and 65 consecutive cases of NHL studied with a range of monoclonal antibodies of pan T cell and T cell subset specificity, other monoclonals of defined specificity for lymphoid cell surface antigens, and "conventional" surface phenotyping of surface immunoglobulin bearing cells (SIg), cytoplasmic immunoglobulin bearing cells (CyIg), E rosetting cells and Fcγ, Fcμ, C3b and C3d rosetting cells in the same samples.

The investigation aimed to (a) assess the specificity, reproducability and immunodiagnostic utility of a number of comparable T cell-specific monoclonals; (b) explore the nature of the T cell components found in association with B cells in the T cell-predominant phase of lymphomas of follicular and of immunoblastic subtypes; (c) establish phenotypic criteria to distinguish T cell-predominant B cell lymphomas from true

Correspondence: J.A. Habeshaw.
Received 27 September 1982; accepted 23 November 1982.

© The Macmillan Press Ltd., 1983.

lymphomas of mature T lineage cells and (d) characterise cells previously described in tumours as "null" or "receptor silent" with "conventional" modes of surface phenotyping.

### Patients

Patients attended the Medical Oncology Unit, St. Bartholomew's Hospital, from January 1981 to December 1981. (Adult patients were under the care of Dr T.A. Lister, and children under that of Professor J.S. Malpas).

### Materials and methods

*Lymph nodes and tissues: cell suspensions*

Single cell suspensions from lymph node biopsies were prepared in tissue culture medium RPMI 1640 within 20 min of surgical removal. Peripheral blood and splenic lymphocytes were prepared by centrifugation on Lymphoprep (Nygaard) at 400 g for 25 min at 20°C. Pleural aspirates were diluted with tissue culture medium RPMI 1640 containing heparin (10 units $ml^{-1}$) and the cells recovered after washing and centrifugation. Cell counts were adjusted to give a concentration of $5 \times 10^6$ nucleated cells $ml^{-1}$ and viability was assessed by trypan blue dye exclusion.

*Preparation of red cells*

Sensitised red cells for assessment of Fc$\gamma$, Fc$\mu$, C3d receptors were prepared as previously described (Habeshaw *et al.*, 1979). C3b-sensitised ox RBC were prepared using R3 reagent incubated with IGM-sensitised ox RBC for 75 sec at 37°C, followed by dilution and washing with ice-cold veronal buffered saline at 0°C. Cells sensitised in this manner are equivalent in their reactivity with C3b receptor expressing cell lines as red cells prepared with purified C components known to express human C3b.

*Preparation and use of antibodies for suspension phenotyping*

For the detection of surface Ig on untreated or acetate washed lymphoid cells in suspension, conventional, commercially available rabbit or goat antisera, or FITC conjugates of IgG preparations, were used. These included antisera against $\mu$, $\gamma$, and $\alpha$ heavy chains, $\kappa$ and $\lambda$ light chains (Nordic, Miles, Dako, Behringwerke). Purified IgG fractions were prepared from whole antiserum by ammonium sulphate precipitation, DEAE sephadex chromatography, or affinity chromatography on staph protein A-Sepharose (Pharmacia). Two preparations of anti-IgD antiserum were used, goat anti-human IgD (courtesy of G. Janossy and S. Mattingley, Royal Free Hospital) and sheep anti-human IgD (Seward Laboratories). $\gamma$-globulin fractions prepared from these antisera were absorbed against CNBR-sepharose coupled human IgG and conjugated with FITC by the method of Forni (1979) to give a final product containing 0.25 mg $ml^{-1}$ antibody and F:P ratio of 2:1 to 4:1. These antisera were diluted to 1:10–1:16, aliquoted in 10 $\mu$l quantities and used at a final dilution of 1:20. All antisera were deaggregated by centrifugation (110,000 g for 1 h) before use.

*Monoclonal antibodies*

The monoclonal antibodies (obtained from commercial sources, or from other laboratories) used in this study are given in the Appendix. They were tested for reactivity against a range of normal lymphocytes from tonsil, blood and lymph nodes, and against CLL or ALL cells where appropriate. Effective dilutions were established empirically for these reagents, which were then aliquoted in 5 $\mu$l quantities and stored as recommended by the manufacturers (at +4°C or below −4°C). The effective antibody concentrations used were from 100–250 $\mu$g $ml^{-1}$ antibody protein and reagents at this concentration were used at final dilutions of 1:15–1:25.

Binding of monoclonal antibodies was detected using goat anti-mouse (GAM) Ig obtained by affinity chromatography of whole goat anti-mouse serum on mouse IgG columns. The retained and eluted Ig was then passed through a column of Sepharose coupled pooled human Ig (IgG and IgM) to remove any cross-reactivity with human Ig. The GAM IgG was coupled with fluorescein isothiocyanate (Sigma) by the method of Forni (1979) to give a product containing 0.38 mg $ml^{-1}$ IgG, with an F:P ratio of 3:1. This preparation was diluted 1:25, aliquoted in 5 $\mu$l quantities, and used at a final dilution of 1:10. Control incubations indicated no detectable reactivity with human leukocyte preparations at the dilutions used.

*Peanut lectin staining*

FITC-conjugated PNA was prepared according to the method described in Rose *et al.* (1980).

*Phenotypic analysis*

The methods of quantitating E rosettes, Fc$\gamma$, Fc$\mu$, C3b and C3d rosettes, SIg and CyIg staining were as previously described (Habeshaw *et al.*, 1979).

Peanut lectin binding was assessed by the method of Rose *et al.* (1981). Staining with monoclonal antibodies was detected by a sandwich technique as follows: The concentration of the cell suspension was adjusted to give $1 \times 10^7$ cells $ml^{-1}$. Fifty-$\mu$l of cell suspension was added to a 500 $\mu$l polypropylene tube containing 5 $\mu$l of the appropriate monoclonal antibody, mixed and incubated for 30 min at 4°C. The cell suspension was washed $\times 2$ in PBS and the supernatant aspirated completely from the cell pellet. The appropriate FITC-conjugated GAM-IgG in 5 $\mu$l aliquots was diluted to 25 $\mu$l in PBS, and added to the cell pellet. Cells were resuspended and incubated for a further 0.5–1 h at 4°C. The stained cells were then washed twice in PBS, pelleted and resuspended in 10 $\mu$l PBS. Wet preparations were made, the coverslips sealed with wax and counted using the Zeiss photomicroscope IV equipped for incident illumination (for fluorescence) and phase contrast microscopy. Viable nucleated cells showing distinct positive fluorescence only were counted. Control preparations included suspensions stained with inappropriate monoclonal antibody (e.g. anti-platelet antibody AN51 (see **Appendix**) or mouse ascites fluid (negative controls). Staining with monoclonal W6/32 (antimonomorphic HLA, A, B, C) and 26/114 (MasO18b Seralab) (anti-$\beta_2$M) were used as positive controls.

### Results

### *I Specificity and reproducability of monoclonal antibody reactions with normal and neoplastic tissues*

The monoclonal antibodies against T cells and T cell subsets gave equivalent results when tested against suspensions of cells from normal tonsil (Table I) and lymph nodes (Table II). There were no significant differences in the proportions of T cells determined by αLeu-I and OKT3 pan T cell antibodies, or between the subset specific monoclonals OKT4/αLeu-3a and OKT8/αLeu-2a. The T cell subset ratios with these pairs of antibodies were not significantly different.

The anatomical distribution of the T cell subsets defined by these monoclonals and B cell types defined by conventional antisera and HLA-Dr specific monoclonals are given in Table III and in Figure 1a–d for normal lymph node tissues.

**Table I** Phenotype analysis of tonsil suspensions with the monoclonal antibody panel

| *E rosette* | *OKT1* | *OKT4* | *OKT6* | *OKT8* | *αLeu-I* | *αLeu-2a* | *αLeu-3a* | *DA-2* | *SIg* | *C3d* | *PNA* |
|---|---|---|---|---|---|---|---|---|---|---|---|
| 30<br>(11–42) | 42.3<br>(37–50) | 29.5<br>(22–37) | 0<br>(0) | 9<br>(6–12) | 38.75<br>(35–46) | 9.75<br>(6–12) | 26.25<br>(22–35) | 50.75<br>(48–54) | 48<br>(41–62) | 45<br>(28–60) | 21.75<br>(10–29) |

Subset ratio OKT/OKT8 = 3.30 αLeu-3a/αLeu-2a = 2.69.
(Mean % values and range in parenthesis for 5 samples are given.)

**Table II** Phenotype analysis of reactive lymph node suspensions by the monoclonal antibody panel, rosette formation, and functional assays

| *Pan T cell markers* | | *T subset markers* | | *Subset ratios* | *Other markers* | |
|---|---|---|---|---|---|---|
| E rosettes | 42.2 ± 2.1<br>(36–55) | OKT4 | 30.9 ± 6.5<br>(12–54) | T4/T8 2.13<br>(0.9–4.2) | SIg$^+$ | 35.2 ± 5.0<br>(18–76) |
| αLeu-I | 58.8 ± 6.0<br>(41–69) | αLeu-3a | 28.0 ± 6.0<br>(11–61) | αLeu-3a/ 2.15<br>αLeu-2a (0.7–5.8) | DA-2<br>(HLA-DR) | 30.56 ± 4.5<br>(18.51) |
| OKT3 | 58.0 ± 5.2<br>(31–78) | OKT8 | 17.7 ± 3.4<br>(9–30) | αLeu-2a/ 2.15<br>OKT4 (0.7–4.5) | Phago-<br>cytes | 8.27 ± 1.9<br>(1–18) |
| UCHTI | 48.4 ± 4.0<br>(37–56) | αLeu-2a | 15.6 ± 2.3<br>(6–21) | αLeu-3a 2.03<br>OKT8 (0.7–4.7) | C3d<br>rosettes | 32.3 ± 4.6<br>(6–60) |
| T4 + T8 | 48.7 ± 5.1<br>(30–67) | | | | C3b<br>rosettes | 11.2<br>(1–20) |
| αLeu-3a<br>+<br>αLeu-2a | 43.6 ± 4.1<br>(27–72) | | | | Fcγ<br>rosettes | 6.8<br>(2–18) |

(Values given are mean % of positive cells, ± s.d., and range of values (in parenthesis)).
(data for W6/32, J5 and OKT6 not shown).
(data for Fcμ and PNA not shown).

**Table III** Location and phenotype of the principle cellular populations of reactive lymphoid tissue assessed on suspensions and frozen sections

| *Cell type* | *Location* | *Phenotype* |
|---|---|---|
| T helper/inducer | Interfollicular area$^+$ germinal centre (sub coronal) | $(E^+)T4^+$ $Leu3a^+$, $T1^+$ $UCHTI^+$, $T3^+$ |
| T suppressor/cytotoxic | T cells and interfollicular zones | $T1^+$ $T3^+$ $UCHTI^+$ $Leu\text{-}2a^+$ |
| B cell of lymphocyte mantle | Lymphocytic mantle of corona | $SIgM^+D^+$, $C3d^+$ DA-2 ($HLA\text{-}Dr^+$) $PNA^-$ |
| B cell of $PNA^+$ type | Germinal centre | SIgG or $A^+$, $C3d^+$ DA-2 ($HLA\text{-}Dr^+$) $PNA^+$ |
| B cell of $PNA^-$ type | Germinal centre | $SIgM^+$, $C3d^\pm$ DA-2 ($HLA\text{-}Dr^+$) PNA- |
| Tingible body macrophage | Germinal centre | $OKT4^+$ $DA\text{-}2^+$ $C3b^+$ $Fc\gamma^+$ |
| Dendritic reticulum cell | Germinal centre | $DA\text{-}2^+$ ($Fc\gamma^+$). IgG and IgM in section. |
| Interdigitating reticulum cell | T cell areas | DA-2 ($HLA\text{-}Dr^+$) |
| Sinusoidal macrophages | Sinuses | $DA\text{-}2^+$ (weakly) $Fc\gamma^+$ $C3b^+$ $OKT8^+$ (in section) $OKT6^+$ (sometimes in section) |

**Figure 1a–d** Normal distribution of T and B subsets in reactive nodes by immunoperoxidase staining.

**Figure 1a** Reactive follicle showing characteristic "margination" of Leu3a + T helper cells to the interface between the corona and germinal centre proper. Primary antibody antibody Leu–3a. Second antibody affinity-purified HRP-labelled goat anti-mouse IgG. ×60.

**Figure 1b** T cell area showing marked positivity for HLA-DR in the interdigitating reticulum cells. Primary antibody DA/2. Second antibody affinity-purified HRP-labelled goat anti-mouse IgG $\times 60$.

**Figure 1c** T cell area showing cellular specificity of staining with the monoclonal antibody $OKT_3$. Primary antibody $OKT_3$. Second antibody affinity-purified HRP-labelled goat anti-mouse IgG $\times 60$.

**Figure 1d** The distribution of $IgD^+$ cells in the coronal zone of normal germinal follicle. Primary antibody goat α human IgD. Second antibody HRP-labelled $F(ab)_2$ rabbit anti-goat IgG ×60.

**Figure 1e** The retention of coronal localization of IgD positivity in follicular lymphoma (case PA, Table VIII). Primary antibody goat anti-human IgD. Second antibody HRP-labelled $F(ab)_2$ rabbit anti-goat Ig ×60.

**Figure 1f** Patient A.S. C-ALL antigen distribution staining with J5 monoclonal clearly limited to the neoplastic follicle. This appearance is typical of follicular lymphoma, and is not seen in normal (reactive) germinal centres. Primary antibody J5 monoclonal against C-ALL antigen. Second antibody HRP-labelled affinity-purified goat anti-mouse IgG ×60.

*II General analysis of the cellular composition of selected classes of malignant lymphoma*

The subset specificity of the monoclonal anti-T cell antibodies was likewise assessed on 7 cases of centroblastic centrocytic follicular lymphoma (Table IV), 7 of immunoblastic lymphoma (Table V) and 5 of malignant lymphoma of lymphocytic type (Table VI). Discrepancies between the reactivity of the pan T cell antibodies αLeu-I and OKT3 were observed in all instances, showing that αLeu-I antibody detects an additional subset of cells expressing the 65K MW glycoprotein antigen, which is not represented in the T cell subset as defined by the OKT3/UCHT-I monoclonals, the sum of OKT4 and OKT8 positive cells, or the $E^+$ populations. These conclusions are compatible with published data on peripheral T cell subsets in man (Reinherz *et al.*, 1979*a*, b, 1980*b*; Janossy *et al.*, 1980; Poppema *et al.*, 1981).

In view of these data, T cell subset-specific monoclonals OKT1/αLeu-I, OKT3/UCHT-I, and the T cell marker E rosettes, are expressed separately, and T cell proportions assessed as defined below.

The total numbers of T cells in a given sample of normal or malignant peripheral lymphoid tissue are equivalent to the population defined by the pan T cell monoclonals OKT3 and UCHT-I. The cytotoxic/suppressor subset is defined as the (Leu-$2a^+$ $OKT8^+$ $OKT3^+$) population, and the helper/inducer subset has the phenotype ($OKT3^+$ $OKT4^+$/Leu-$3a^+$). In the examination of the individual lymph nodes in cases of malignant lymphoma (Section III below), the phenotypic groupings used are (Leu-$I^+$), (OKT3/UCHT-$I^+$), (OKT4/Leu-$3a^+$) and (OKT8/Leu-$2a^+$), corresponding to total T cells (+non-T cells), total T cells, T "helper/inducer" and T "cytotoxic/suppressor" subclasses.

*III Individual case analysis of phenotype in NHL*

The conclusions from the preliminary studies (**Results**: section I), influenced the presentation and interpretation of data in this section. The values for αLeu-I are presented separately from the values of OKT3 and UCHT-I positive cells. Since values obtained for $OKT4^+$ and Leu-$3a^+$ cell populations appeared identical, the mean values ($OKT4^+$) plus (Leu-$3a^+$) for helper/inducer subsets are presented. The mean values for ($OKT8^+$) and (Leu-$2a^+$) cells similarly represent the proportions of cytotoxic/suppressor T cell subsets. The "helper/suppressor" ratio is presented as $OKT4^+$/$OKT8^+$ cells. Data obtained with the monoclonals W6/32 (anti-HLA ABC), 26/114 (anti-$\beta_2$M) and 2DI (anti-HLeI) are shown or discussed only where relevant. The monoclonal AN51 and

**Table IV** Phenotypic analysis of 7 cases of centroblastic/centrocytic follicular lymphoma by the monoclonal antibody panel and other cell surface markers

| *Pan T cell markers* | | *T subset markers* | | *Subset ratios* | | *Other markers* | |
|---|---|---|---|---|---|---|---|
| E rosettes | 17.1±4.9 (6–42) | OKT4 | 12.29±3.2 (3–23) | OKT4/OKT8 | 2.28 (0.2–5.8) | SIg$^+$ B cells | 50.0±4.5 (31–72) |
| αLeu-I | 30.0±0.8 (27–32) | αLeu-3a | 14.4±3.1 (3–21) | αLeu-3a/ αLeu-2a | 3.26 0.43–6.33) | HLA-DR$^+$ cells | 33.7±6.1 (24–62) |
| OKT3 | 24.9±2.5 (14–31) | OKT4 | 12.0±3.0 (4–29) | | | "Null" cells | 12.9±3.4 (4–42) |
| UCHTI | 18.5±3.3 (11–26) | αLeu-3a | 7.6±2.3 (3–17) | | | C3d receptor | 22.0±5.2 (0–24) |
| OKT4+OKT8 | 25.7±2.5 (19–35) | | | | | C3b receptor | 7.6±3.4 (1–10) |
| αLeu-2a + αLeu-3a | 19.6±3.5 (10–29) | | | | | Fcγ receptor | 7.6±4.4 (1–10) |
| | | | | | | PNA | 29.4±7.3 (1–50) |
| | | | | | | J5$^+$ cells | 7.0±4.0 (0–16) |

Values are included for Null cells, and for J5 positive cells.
(data for W6/32 and OKT6 not shown).

**Table V** Phenotypic analysis of lymph node suspensions from 7 cases of immunoblastic malignant lymphoma by the monoclonal antibody panel and the other cell surface markers

| *Pan T cell markers* | | *T subset markers* | | *Subset ratios* | | *Other markers* | |
|---|---|---|---|---|---|---|---|
| E rosettes | 38.8±7.6 (14–73) | OKT4 | 24.0±10.4 (2–61) | OKT4/OKT8 | 1.38 (0.3–4.36) | SIg$^+$ B cells | 32.3±8.4 (3.72) |
| αLeu-I | 63.8±15.5 (10–91) | αLeu-3a | 22.6±8.6 (3–49) | αLeu-3a/ αLeu-2a | 1.80 (0.38±4.45) | HLA-Dr cells (DA-2) | 31.5±9.5 (3–58) |
| OKT3 | 52.0±13.4 (18–92) | OKT8 | 18.7±7.4 (2–48) | | | Null cells | 11.4±6.2 (0–42) |
| UCHTI | (2 values only) (8, 70) | αLeu-2a | 13.6±3.1 (8–22) | | | C3d receptor | 11.4±5.2 (1–38) |
| OKT4$^+$ OKT8 | 42.7±11.8 ( 4–75) | | | | | C3b receptor | 2.4±0.9 (0–6) |
| αLeu-2a$^+$ αLeu-3a | 36.0±9.6 (11–60) | | | | | Fcγ receptor | 7.9±4.8 (1–30) |
| | | | | | | Fcμ receptor | 2.5±1.4 (0–11) |
| | | | | | | PNA | 21.4±6.1 (8–55) |

(data for W6/32, J5 and OKT6 not shown).
The values given are the mean % of cells positive for each marker ±s.d., and range of values (in parenthesis).

**Table VI** Phenotype analysis of lymph node suspensions from 5 cases of malignant lymphoma of lymphocytic type by the monoclonal antibody panel and other cell surface markers

| *Pan T cell markers* | | *T subset markers* | | *Subset ratios* | | *Other markers* | |
|---|---|---|---|---|---|---|---|
| E rosettes | 31.3 ± 15.6 (3–70) | OKT4 | 15.8 ± 5.5 (8–35) | OKT4/OKT8 | 1.32 (0.5–4.38) | SIg$^+$ B cells | 35.4 ± 13.1 (4–71) |
| αLeu-I | 39.8 ± 10.6 (25–73) | αLeu-3a | 11.3 ± 5.3 (3–24) | αLeu-3a/ αLeu-2a | 1.32 (0.23–2.33) | HLA-DR$^+$ cells | 42.0 ± 11.5 (15–73) |
| OKT3 | 27.8 ± 6.7 (12–40) | OKT8 | 16.0 ± 4.7 (5–28) | | | "Null" cells | 33.4 ± 12.4 (4–73) |
| OKT4 + OKT8 | 32.2 ± 6.2 (13–43) | αLeu-2a | 18.0 ± 5.4 (7–30) | | | C3d receptor | 29.8 ± 10.7 (1–56) |
| αLeu-2a + αLeu-3a | 29.3 ± 6.7 (10–41) | | | | | C3b receptor | 16.2 ± 7.5 (4–42) |
| | | | | | | Fcγ receptor | 19.0 ± 11.8 (1–58) |
| | | | | | | Fcμ receptor | 15.2 ± 9.7 (1–57) |

The values given are the mean % of cells positive for each marker, ± s.d. and the range of values (in parenthesis).
(data for W6/32, OKT6 and UCHTI (2 only) not shown)
Data for PNA (negative), J5 (negative) are excluded.

mouse ascitic fluid gave negative reactions in all the presented cases.

The data in this section are presented according to the histopathological diagnosis based on Lennert's modification of the Kiel classification. The largest group (tumours of follicular derivation) are presented first. These include centroblastic malignant lymphoma (Table VII), centroblastic and centrocytic follicular lymphoma (Table VIII) and centrocytic lymphoma of small or large cell type (Table IX). Tumours of follicular centre cell derivation are phenotypically classified as being of B cell origin, despite the sometimes high, and occasionally predominant, populations of T cells within the lesions.

Two of the 5 cases of *centroblastic lymphoma* (I.C. and C.B.) showed T cell predominance as assessed by presence of (OKT3$^+$ UCHT-I$^+$ Leu-I$^+$) T cells.

All cases showed light chain class restricted B cell populations of variable heavy chain isotype. Patient I.C. had, in addition, a population of light chain class restricted plasma cells not expressing HLA-DR antigen. Patients C.B. and J.S. showed significant populations of OKT9$^+$ cells, which might reflect the comparatively large proportion of proliferating cells in this class of tumour. PNA and C3d receptor expression do not appear to be important markers of B cell populations in centroblastic lymphoma.

The 15 patients with *centroblastic centrocytic follicular lymphoma* (Table VIII) show patients A.B., M.W., A.Br., E.Z., J.K.N. and A.D. with T cell predominance. In patients J.K.N., A.D., A.Br., the OKT3$^+$ UCHT-I$^+$ cells were less than the sum of the OKT4$^+$ OKT8$^+$ populations, suggesting the presence in these lesions of OKT8$^+$/OKT4$^+$ double-marked T cells, or alternatively, the expression of OKT4$^+$ on an additional non-T populations. Double staining was not done in these cases.

Patients P.A., M.W., M.E. and J.K.N. showed an excess of Leu-I$^+$ cells over total T cells (as defined by OKT3$^+$ UCHT-I$^+$, and sum of (OKT4$^+$) and (OKT8$^+$) cells), suggesting αLeu-I presence on non-T populations. Patient D.C. showed a marked excess of cytotoxic/suppressor T cells (OKT8$^+$) over T helper/inducer cells (OKT4$^+$), T4/T8 ratio 0.07. In patients P.A. and M.E. the T cell population was E$^-$. In 7 patients (D.C., M.E., M.W., A.Br., J.K.N., S.A., A.D.) the B cell population did not show light chain class restriction (by established criteria of suspension phenotyping K:L ratio $>10$, L:K ratio $>5$). The values obtained for PNA positivity and C3d receptor expression show the heterogeneity of follicular B cell subsets; in 6 cases both C3d and PNA are expressed (A.S., R.N., M.W., E.B., A.M., J.K.N.), and in 3 cases neither is expressed (A.D., P.A., A.Br). These markers are concordant in the

**Table VII** Phenotype of lymph node suspensions from patients with centroblastic lymphoma (MLCB)

| | *Patient Age/Sex Histology* | | | | |
|---|---|---|---|---|---|
| | *CB 50/M MLCB/PO* | *JS 43/M MLCB* | *HT 49/F MLCB* | *MB 68/M MLCB* | *IC 50/F MLCB/PO* |
| E | 37 | 7 | 11 | 20 | 29 |
| Fcγ | 2 | 0 | 3 | 10 | 2 |
| Fcμ | 0 | 0 | 0 | 0 | 9 |
| C3b | 0 | 3 | 0 | 11 | 0 |
| C3d | 1 | 12 | 34 | 10 | 28 |
| G | 1 | 4 | 16 | 2 | 17 |
| M | 1 | 78 | 60 | 60 | 10 |
| A | 30 | 0 | 4 | 4 | 6 |
| D | 3 | 0 | 4 | 0 | 0 |
| κ | 22 | 2 | 3 | 50 | 49 |
| λ | 2 | 69 | 65 | 3 | 1 |
| PNA | 2 | 0 | 0 | 3 | 10 |
| DA-2 | 71 | 82 | 70 | 46 | 19 |
| J5 | 0 | 6 | 0 | 0 | 0 |
| αLeu-I | 63 | 20 | 14 | 6 | 70 |
| OKT3/UCHTI | 75 | 25 | 15 | 8 | 67 |
| OKT4/αLeu-3a | 49 | 5 | 7 | 4 | 21 |
| OKT8/αLeu-2a | 17 | 15 | 6 | 7 | 35 |
| OKT9 | 18 | 22 | 8 | 6 | 7 |
| OKT6 | 4 | 0 | 0 | 0 | 0 |
| CyIg | Neg | ND | Neg | Neg | 10%K* |
| T4/T8 ratio | 2.9 | 0.3 | 1.1 | 0.57 | 0.60 |

*CyIg positivity is only noted where >10% of the cells in the lesion are CyIg positive and light chain class restricted.
MLCB/PO = Malignant lymphoma, centroblastic; polymorphic subtype.

majority of follicular lymphomas. In 8/13 tested cases the monoclonal J5 reacted with >10% of cells, and J5 staining was restricted to B cells in suspension (an observation subsequently confirmed by double staining and in frozen section). (Figure 1f) Patient A.M., with a transforming lesion (CBccF→CB/cc/D) showed low numbers of T cells, but high OKT9 expression. Patients E.Z. and A.D., also transforming, showed T cell predominance, but low numbers of OKT9$^+$ cells. Thus the histological assessment of transformation includes 2 different phenotypic entities, one of T cell predominance, and one of B cell predominance with increased expression of trf receptor.

Six cases of centrocytic lymphoma (Table IX) showed a B cell predominant phenotype, with low levels of total T cells. In patients A.C. and G.Co., Leu-I expressing cells were more frequent than total T cells. All patients except G.C. showed monoclonal B cell phenotype. Patient G.C. showed a "null" cell phenotype with strong HLA-DR expression on most cells. It is of interest that the B cells in the

**Table VIII** Phenotype of lymph node suspensions from patients with centroblastic and centrocytic lymphoma (CB/cc/F)

| | *Patient Age/Sex* | | | | | | | | | | | | | | |
|---|---|---|---|---|---|---|---|---|---|---|---|---|---|---|---|
| | *AS* 23/*F* | *PA* 52/*M* | *DC* 47/*M* | *RN* 56/*F* | *ME* 44/*F* | *MW* 47/*M* | *EB* 57/*F* | *ABR* 56/*F* | *JP* 33/*F* | (*T*) *EZ* 64/*F* | *MG* 58/*M* | (*T*) *AM* 69/*F* | *JKN* 35/*F* | (*Spl*) *SA* 51/*F* | (*T*) *AD* 62/*M* |
| Markers | | | | | | | | | | | | | | | |
| E | 23 | 1 | 12 | 27 | 4 | 38 | 8 | 65 | 69 | 51 | 2 | 1 | 54 | 59 | 18 |
| Fcγ | 4 | 10 | 48 | 0 | 1 | 12 | 0 | 1 | 5 | 36 | 2 | 11 | 2 | 41 | 11 |
| Fcμ | 2 | 1 | 0 | 0 | <1 | 0 | 0 | 2 | 1 | 4 | 0 | 2 | 0 | 5 | 0 |
| C3b | 24 | 6 | 0 | 0 | 6 | ND | 2 | 1 | 0 | 0 | 0 | 12 | 1 | 1 | 1 |
| C3d | 29 | 1 | 30 | 42 | 26 | 28 | 33 | 11 | 3 | 11 | 3 | 31 | 19 | 33 | 4 |
| G | 1 | 7 | 0 | 24 | 6 | 5 | 32 | 24 | 14 | 15 | 74 | 6 | 14 | 31 | 27 |
| M | 31 | 13 | 35 | 12 | 16 | 14 | 60 | 2 | 8 | 1 | 3 | 90 | 29 | 28 | 1 |
| A | 0 | 3 | 37 | 2 | <1 | 21 | 3 | 12 | 26 | 1 | 13 | 3 | 6 | 18 | 4 |
| D | 1 | 11 | 45 | 11 | 0 | 12 | 22 | 0 | 14 | 2 | 0 | 23 | 10 | 5 | 4 |
| κ | 3 | 34 | 30 | 30 | 21 | 36 | 62 | 14 | 3 | 17 | 5 | 10 | 12 | 14 | 47 |
| λ | 28 | 4 | 40 | 3 | 6 | 8 | 13 | 16 | 20 | 4 | 3 | 70 | 7 | 36 | 8 |
| PNA | 50 | 74 | ND | 21 | 0 | 26 | 56 | 4 | ND | 2 | 72 | 80 | 11 | ND | 0 |
| J5 | 16 | 2 | 16 | ND | 6 | 12 | 30 | 3 | 14 | 5 | 27 | 34 | 12 | 0 | ND |
| DA-2 | 26 | 24 | ND | 24 | 50 | 12 | 55 | 45 | 30 | 13 | 1 | 11 | 46 | 6 | 2 |
| αLeu-I | 31 | 31 | 28 | 27 | 49 | 71 | 21 | 58 | 42 | ND | 13 | 2 | 93 | 33 | 35 |
| OKT3/UCHT-I | 31 | 19 | 31 | 30 | 40 | 60 | 22 | 68 | 53 | 88 | 8 | 4 | 68 | 39 | 54 |
| OKT4/αLeu-3a | 22 | 21 | 2 | 10 | 20 | 48 | 15 | 45 | 28 | 43 | 9 | 1 | 62 | 18 | 50 |
| OKT8/αLeu-2a | 10 | 4 | 29 | 9 | 10 | 12 | 6 | 50 | 22 | 12 | 5 | 3 | 16 | 17 | 16 |
| OKT9 | 2 | <1 | ND | ND | 0 | 2 | 1 | 3 | 5 | 4 | 2 | 60 | 4 | 0 | 0 |
| SIg class | ML | MD/K | Pcl | GK | Pcl | Pcl | G/MD/K | Pcl | G,A,L | GK | G | ML | Pcl | Pcl | Pcl |
| OKT4/OKT8 ratio | 2.2 | 5.3 | 0.07 | 1.1 | 2.0 | 4.0 | 2.5 | 0.92 | 1.27 | 3.6 | 1.8 | 0.3 | 4.13 | 1.1 | 3.1 |

(T) = centroblastic and centrocyclic follicular lymphoma "transforming".
(Spl) = phenotypic profile of involved spleen.

pleural effusion from patient W.McK do not reflect the monoclonality of the lymph node population, or the blood B cells. One patient (A.C.) showed 22% $OKT9^+$ cells, but in all other cases $OKT9^+$ cells were infrequent. Two of the centrocytic large cell tumour patients, W.McK. and G.Co., had only IgM heavy chain isotype expressed on the B cells. In the patients with centrocytic tumours of small cell type, heavy chain isotype expression was more mixed, including G- and A-positive cells. Only one patient, P.C., showed a significant proportion of IgD-bearing cells, in contrast to the patients with follicular lymphoma.

### *Immunoblastic lymphoma*

Eleven patients with immunoblastic malignant lymphoma had phenotypic profiles of involved tissues (Table X). In patients G.S., J.P. and T.McG. the histological appearances were those of "T cell" immunoblastic lymphoma. In patient G.S., T cells with Leu-I/$OKT3^+$ phenotype were present in excess of the $T4^+$ and $T8^+$ subclasses. In double-staining reactions a small $SIg^+$ population (1–2%) was $OKT3^+$. The profile of T.McG. was similar with a marked overlap of OKT3, Leu-I and κ chain staining on the blast-transformed population. On subsequent cloning and culture these cells lost OKT3 positivity, reverting to G κ positive B cells in 5 weeks (Izaguirre, 1982). Patient J.P. showed a strongly κ chain-predominant B cell component in the lesion.

Patients P.C. and J.M. showed an excess of αLeu-I expressing cells over total T cells. In P.C. and J.M. a clear overlap of this αLeu-I marker and SIg positivity was seen. In patient J.M. 28% of cells were $J5^+$, the only immunoblastic lymphoma to show this pattern. In patient J.E., αLeu-I was found on a major $SIg^-$ population. Patients J.E. and J.M. also showed some PNA positivity, more usually a marker of follicular lymphoma.

Three patients showed substantial populations of $CyIg^+$ B cells, including the two T cell predominant immunoblastic lymphomas of G.S. and T.McG. In patient D.B., only κ chain staining plasma cells/plasmablasts were found, despite the presence

**Table IX** Phenotype of lymph node suspensions from patients with centrocytic lymphoma

| | *Patient Age/Sex Histology* | | | | | | | |
|---|---|---|---|---|---|---|---|---|
| | *AC* 60/*M* *MLccSc* | *WMcK(N)** 68/*M* *MLccLc* | *WMcK(PE)** 68/*M* — | *WMcK(B)** 68/*M* *LSCL* | *GC* 27/*F* *MLccLc* | *GCo* 62/*M* *MLccLc* | *DL* *M* *MLccSc* | *PC* 55/*M* *MLccSc* |
| Markers | | | | | | | | |
| E | 2 | 2 | 42 | 19 | 3 | 20 | 6 | 14 |
| Fcγ | 0 | <1 | <1 | 1 | 2 | 1 | 0 | ND |
| Fcμ | 0 | <1 | <1 | <1 | 0 | 0 | 0 | ND |
| C3b | ND | 3 | ND | <1 | 0 | 4 | 12 | ND |
| C3d | 8 | 6 | ND | <1 | ND | 42 | 3 | ND |
| G | 19 | 30 | 8 | 6 | 1 | 20 | 16 | 70 |
| M | 83 | 47 | 8 | 53 | 0 | 69 | 5 | 80 |
| A | 16 | 0 | 4 | 16 | 2 | 2 | 0 | 10 |
| D | 3 | 0 | 1 | 0 | 0 | 0 | 4 | 37 |
| κ | 12 | 0 | 21 | 4 | 2 | 2 | 60 | 84 |
| λ | 85 | 70 | 15 | 45 | 3 | 76 | 3 | 19 |
| PNA | 0 | 0 | 0 | 4 | 0 | 6 | 0 | 0 |
| DA2 | 37 | ND | 3 | 76 | 46 | 71 | 47 | 29 |
| J5 | 0 | 0 | 0 | ND | 0 | 0 | 0 | 0 |
| αLeuI | 60 | ND | 66 | 10 | 4 | 40 | ND | 32 |
| OKT3/UCHTI | 4 | ND | 54 | 11 | 8 | 20 | 30 | 14 |
| OKT4/αLeu-3a | 1 | ND | 32 | 6 | 1 | 16 | 11 | 4 |
| OKT8/αLeu-2a | 4 | ND | 8 | 6 | 2 | 3 | 10 | 12 |
| OKT9 | 22 | 0 | 0 | 4 | 1 | 0 | 0 | 0 |
| OKT6 | 0 | 0 | 0 | 3 | 0 | 0 | 1 | 2 |
| CyIg | Neg | Neg | ND | ND | Neg | Neg | Neg | Neg |
| T4/T8 ratio | 0.25 | — | 4.0 | 1.0 | 0.5 | 5.3 | 1.1 | 0.3 |

*N = Node, PE = Pleural Effusion, B = Blood.

of both κ- and λ-staining B cells in the lymph node suspension. Six patients clearly showed monoclonal SmIg (P.C., G.C., J.M., E.H., A.L., T.McG.). In patients D.B. and G.S., CyIg expression was of one light chain class. Patient J.E. lacked $SIg^+$ cells, and patients J.P. and F.O'C. had large excesses of κ chain staining cells which were not nominally monoclonal (as defined by κ:λ ratios).

A striking feature of these immunoblastic lymphomas is the extent of OKT9 positivity found in the neoplastic population: in 2 cases, D.B. and E.H., the majority of cells present expressed trf receptors.

In view of the data from surface phenotyping of immunoblastic lymphoma and follicular lymphoma, the status of the entity "T cell immunoblastic" lymphoma is not clear. T cell predominance in a monoclonal B cell tumour, in which the T cells have undergone blast transformation, or in which a B cell subset expresses acquired or dysfunctional OKT3 markers, is superficially a "T" cell lymphoma (at least on histological criteria). It is important to recognise that although in immunoblastic lymphoma the blast-transformed population marks as "T" lymphoid, there may be phenotypic evidence of an additional, non-transformed "monoclonal" B cell population.

*Malignant lymphocytic lymphoma*

The profiles of involved tissues of 10 patients with malignant lymphocytic lymphoma, and CLL, are given in Table XI. Patient J.B. was described as having pro-lymphocytic variant of MLL, and on histological grounds the lesion of E.B. showed some features of T-CLL involving lymph node. Six patients (F.B., G.J., L.O'D., J.C., J.B., J.N.) showed major populations of $Leu\text{-}I^+$ cells. In L.O'D. αLeu-I was the only marker expressed. In patients J.C. and G.J., αLeu-I and SIg only were expressed. Patient J.B. had αLeu-I, expressed with HLA-DR ($Leu\text{-}I^+$ $SIg^+$ (? $HLA\text{-}DR^+$). The profile of patient J.N. was $E^+$, $\alpha Leu\text{-}I^+$, $HLA\text{-}DR^+$, but $SIg^-$. The $E^+$, $Leu\text{-}I^+$ cells in patient J.N. lacked the OKT3/UCHT-I pan T cell markers, and also the OKT4/αLeu-3a, OKT8/αLeu-2a subset markers, but was clearly $HLA\text{-}DR^+$. Patient E.B. showed cells of similar phenotype in the peripheral blood (not shown) but there is no evidence of this population in lymph node. One difficulty in phenotyping lymphomas of this class is in the assessment of SIg positivity, by

**Table X** Phenotype of lymph node suspensions from patients with immunoblastic lymphoma

| | *Patient Age/Sex Histology* | | | | | | | | | | |
|---|---|---|---|---|---|---|---|---|---|---|---|
| | *DB* 57/*F* *MLIB* | *PC* 31/*M* *MLIB* | *GC* 47/*M* *MLPHg* | *GS* 59/*M* *MLIB(T)* | *JE* 62/*M* *MLPHg* | *JM* 59/*F* *MLIB* | *JP* 72/*F* *MLIB(T)* | *EH* 67/*M* *MLIB* | *AL* 74/*F* *MLIB* | *FO'C* 68/*M* *MLPHg* | *TMcG* 56/*M* *MLIB(T)* |
| Markers | | | | | | | | | | | |
| E | 25 | 13 | 56 | 36 | 42 | 31 | 18 | 10 | 5 | 25 | 0 |
| Fcγ | 11 | 2 | 30 | 0 | 3 | 0 | <1 | 1 | 46 | 0 | ND |
| Fcμ | 0 | 11 | <1 | 0 | 2 | 2 | 0 | 1 | 29 | 1 | ND |
| C3b | 1 | ND | 0 | 0 | 6 | 4 | 5 | 1 | ND | 0 | ND |
| C3d | 26 | 1 | 16 | 0 | 6 | 1 | 24 | 13 | 12 | 4 | ND |
| G | 20 | 58 | 32 | 0 | 0 | 2 | ND | 2 | 10 | 15 | 91 |
| M | 9 | 8 | 6 | <1 | 8 | 45 | ND | 81 | 38 | 4 | 0 |
| A | 3 | 5 | 0 | 0 | 0 | 9 | ND | <1 | 1 | <1 | <1 |
| D | 2 | 36 | 30 | 0 | <1 | 3 | ND | 80 | 1 | 0 | <1 |
| κ | 27 | 48 | 30 | <1 | 4 | 51 | 23 | 73 | 90 | 33 | 93 |
| λ | 12 | 4 | <1 | <1 | 1 | 1 | 4 | 1 | 2 | 6 | 2 |
| PNA | 1 | 0 | 8 | 6 | 38 | 85 | ND | 2 | 1 | 11 | 5 |
| J5 | 0 | ND | ND | 0 | 1 | 28 | ND | 0 | 0 | 0 | ND |
| DA2 | 76 | 85 | 24 | 3 | 28 | 55 | 21 | 83 | 27 | 36 | 60 |
| αLeuI | 17 | 72 | 35 | 91 | 95 | 85 | 25 | 5 | 8 | 18 | 68 |
| OKT3/UCHTI | 25 | 18 | 62 | 92 | 45 | 64 | 29 | 16 | 2 | 36 | 70 |
| OKT3/αLeu-2a | 15 | 17 | 15 | 44 | 2 | 47 | 12 | 9 | 2 | 12 | 8 |
| OKT8/αLeu-2a | 18 | 10 | 48 | 18 | 5 | 11 | 24 | 9 | 1 | 15 | 0 |
| OKT9 | 58 | 18 | ND | 2 | 19 | 5 | 6 | 51 | 42 | 11 | 5 |
| OKT6 | 0 | 0 | ND | 0 | 0 | <1 | ND | ND | 0 | 0 | 0 |
| CyIg | 10%K+ | Neg | Neg | 11%MK | Neg | Neg | Neg | Neg | Neg | Neg | 30%GK |
| T4/T8 ratio | 0.83 | 1.7 | 0.31 | 2.44 | 0.4 | 4.3 | 0.5 | 1.0 | 2.0 | 0.8 | 8.0 |

MLIB = Immunoblastic Malignant Lymphoma MLP(Hg) = Malignant lymphoma pleomorphic (High grade).
The suffix (T) denotes histological appearances of "T" immunoblastic lymphoma.

immunofluorescence, since SIg is only weakly expressed, and only 5 patients (G.J., J.C., J.B., H.M., E.B.) were thought to show clearly monoclonal B cell populations. Previous observations on the expression of Fcγ, Fcμ receptors (Patients J.B. and E.H.) and C3b and C3d receptors (Patients G.J., E.H., J.N., F.B.) are confirmed here and demonstrate the difference between cells of CLL type and follicular centre B cells in expression of these markers. "Null" cell populations were present in patients E.H., H.M. and L.G. (defined as Non T; SIg-, HLA-DR-populations) although some cytoplasmic IgM κ plasmacytoid cells were present in patient H.M.

*T cell lymphoma in adults*

Only comparatively recently have T cell lymphomas been recognised by a combination of histological and phenotypic criteria, and there are no established rules for classifying lymphomas of T cells phenotypically. In this paper, the following phenotypic criteria are regarded as essential before a lymphoma can be unambiguously described as "T" cell:

1 Lymphoma of lymphoblastic histology with acid phosphatase positivity and TdT positivity composed predominantly of cells expressing 2 or more antigenic markers of T lineage specificity. Lesions fulfilling these criteria are classified as T lymphoblastic lymphoma.
2 Lymphoma of immunoblastic or high grade histology, with cytological features of T cells (convoluted or cerebreform nuclei) lacking TdT enzyme, composed predominantly of cells expressing 2 or more antigenic markers of T lineage specificity, showing predominance of a single T cell subset, and with no evidence of B lymphocyte light chain class restriction.
3 Lymphoma of low grade histology with cytological features of T cells, lacking TdT enzyme, expressing 2 or more antigenic markers of T lineage specificity, showing predominance of a single T cell subset, and with no evidence of B lymphocyte light chain class restriction.

The antigenic markers of T lineage specificity considered are E rosette (or OKT11a) positivity, OKT3/UCHT-I, OKT4/αLeu-3a, OKT8/αLeu-2a

**Table XI** Phenotype of lymph node suspensions from patients with malignant lymphoma of lymphocytic type

| | *Patient Age/Sex Histology* | | | | | | | | | | |
|---|---|---|---|---|---|---|---|---|---|---|---|
| | *LG 65/F MLL(Node)* | *LG 65/F MLL(Spleen)* | *FB 50/M MLL* | *GJ 58/M MLL* | *LO'D 54/F MLL* | *JC 53/M MLL* | *JB 64/M MLL* | *EH 72/M MLL* | *HM 49/F MLL* | *JN 57/M MLL* | *EB 46/F MLL* |
| Markers | | | | | | | | | | | |
| E | 2 | 8 | 11 | 3 | 6 | 5 | 32 | 32 | 3 | 70 | 11 |
| Fcγ | 1 | 15 | 2 | 8 | 9 | 34 | 58 | 23 | 1 | 0 | <1 |
| Fcμ | 6 | 7 | 8 | 16 | 3 | 0 | 16 | 57 | 12 | 0 | <1 |
| C3b | 4 | 5 | 35 | 33 | 0 | 0 | 9 | 16 | 10 | 42 | 4 |
| C3d | 2 | 5 | 55 | 10 | 2 | 8 | 50 | 41 | 0 | 56 | <1 |
| G | 32 | 7 | 37 | 31 | 3 | 5 | 38 | 9 | 10 | 0 | 5 |
| M | 10 | 2 | 3 | 17 | 0 | 23 | 71 | 2 | 13 | 3 | 0 |
| A | <1 | 2 | 4 | 5 | 1 | 1 | 0 | <1 | 0 | 1 | 0 |
| D | 0 | 0 | 1 | 3 | 0 | 2 | 35 | 5 | 0 | 0 | 2 |
| κ | 3 | 2 | 10 | 57 | 2 | 46 | 70 | 9 | 23 | 4 | 88 |
| λ | 4 | 7 | 20 | 1 | 1 | 6 | <1 | 11 | <1 | <1 | 0 |
| PNA | 0 | ND | 0 | ND | 0 | ND | 8 | 18 | ND | 1 | 0 |
| DA2 | 5 | 7 | 56 | 17 | 2 | 8 | 46 | 15 | 24 | 73 | 67 |
| J5 | 0 | 0 | 0 | 0 | 0 | 0 | ND | 0 | 0 | 0 | ND |
| αLeuI | 20 | 37 | 60 | 85 | 56 | 46 | 61 | 25 | ND | 94 | 42 |
| OKT3/UCHTI | 8 | 25 | 10 | 10 | 8 | 12 | 34 | 39 | 11 | 12 | 40 |
| OKT4/αLeu-3a | 8 | 3 | 7 | 4 | 5 | 11 | 13 | 13 | 8 | 8 | 32 |
| OKT8/αLeu-2a | 3 | 15 | 3 | 3 | 4 | 4 | 21 | 17 | 14 | 4 | 8 |
| OKT9 | 0 | 2 | 0 | 0 | 0 | <1 | ND | 0 | 2 | 2 | 4 |
| OKT6 | 0 | 0 | 0 | 0 | 0 | 0 | ND | <1 | 0 | ND | ND |
| CyIg | Neg | Neg | Neg | Neg | Neg | Neg | Neg | Neg | Neg | Neg | Neg |
| T4/T8 ratio | 2.7 | 0.2 | 2.3 | 1.3 | 1.2 | 2.8 | 0.62 | 0.76 | 0.57 | 2.0 | 4.0 |

and OKT6 positivity (valid only for TdT positive lymphomas); OKT-I and Leu-I positivity alone cannot be regarded as T lineage specific, neither can the OKT9 marker. The T helper/inducer-to-T cytotoxic/suppressor ratio is not regarded as sufficient criterion for the determination of T subset predominance, since (a) double-marked (T4$^+$ T8$^+$) T cell populations are known to exist, and (b) the major T cell population may be OKT3/UCHT-I$^+$, but OKT4/Leu-3a$^-$ or OKT8/Leu-2a$^-$. In such instances the "helper/suppressor" ratio is probably irrelevant.

It is also important to recognise that apparent monoclonal (i.e. light chain class-restricted) B cell populations can occur in tumours composed predominantly of T cells, in which both major subsets of T cells are present. In such cases, the correct descriptive term applied to the phenotype is "T cell predominant monoclonal B". Five cases of T cell lymphoma in adults which fulfil these criteria are given in Table XII. An additional case of "T" lymphoma diagnosed on histology/cytology but not fulfilling all these criteria is also shown here (Patient R.E.).

The patients A.S., F.S., E.M., P.J., C.M. (Table XII) with adult "T cell" lymphoma showed T cell predominance in the affected tissues, of a single defined T cell subset. In patient F.S., with classical T zone lymphoma involving lymph node paracortex, this T cell population was phenotypically E$^+$, Leu-I$^+$, OKT3$^+$ and essentially OKT4/Leu-3a$^-$, OKT8/Leu-2a$^-$. In patient A.S., with Sezary cell leukaemia, the phenotype was E$^+$ Leu-I$^+$ OKT4$^+$ OKT8$^+$, with strong suggestion of overlap of these markers on the affected population. In patient C.M., with T cell PLL, the major population was E$^+$ Leu-I$^+$ OKT3$^+$ with a high proportion of OKT9$^+$ cells.

Patient E.M. had Sezary cell leukaemia supervening in long-standing mycosis fungoides. The lymph node showed E$^+$, αLeu-I$^+$, OKT3$^+$, UCHT-I$^+$, OKT4$^+$ and αLeu-3a$^+$ to be the phenotype of the major cell population. More cells were E$^+$ and αLeu-I$^+$ than marked with OKT3/UCHT-I or OKT4. The node lesion was characterised as mature T helper/inducer T cell lymphoma (E$^+$ Leu-I$^+$ OKT3$^+$ OKT4$^+$). In blood a very similar T cell phenotype was found, but here the majority of cells were E$^-$.

Patient P.J. had a high grade diffuse lymphoma of angular rather than convoluted cells showing moderate mitotic activity, with no other obvious features of T cell disease histologically. There was no involvement of skin. The phenotype of this

**Table XII** Lymphomas classified as adult T cell lymphoma by phenotype criteria

| | *Patient Age/Sex* | | | | | | | | |
|---|---|---|---|---|---|---|---|---|---|
| | *AS* 70/*M* (*blood*) | *FS* 64/*M* (*node*) | *EM* 71/*F* (*node*) | *EM* 71/*F* (*blood*) | *PJ* 48/*M* (*node*) | *RE* 63/*M* (*node* 1) | *RE* 63/*M* (*eff.*) | *RE* 63*M* (*node* 2) | *CM* 31/*F* (*blood*) |
| Markers | | | | | | | | | |
| E rosettes | 51 | 75 | 71 | 9 | 10 | 3 | 81 | ND | 83 |
| αLeu-I | 64 | 78 | 91 | 96 | 98 | 23 | 61 | 18 | 57 |
| OKT3/UCHT-I | ND | 83 | 58 | 96 | 92 | 47 | 87 | 11 | 61 |
| OKT4/αLeu-3a | 56 | 5 | 36 | 86 | 95 | 39 | 64 | 16 | 0 |
| OKT8/αLeu-2a | 56 | 1 | 3 | <1 | <1 | 25 | 23 | 2 | 5 |
| OKT9 | ND | ND | 3 | 0 | 0 | 100* | 0 | 100* | 45 |
| OKT6 | ND | 0 | 0 | 0 | 0 | 0 | ND | 0 | 0 |
| DA2 | 8 | <1 | 3 | 4 | 4 | 16 | 3 | <1 | 2 |
| SIg | ND | 10 | 11 | 3 | 5 | 15 | 3 | 8 | 8 |
| Fcγ | 8 | 18 | 2 | 20 | 3 | 0 | 15 | 3 | ND |
| Fcμ | <1 | 0 | 0 | 1 | 0 | 0 | 67 | 8 | ND |
| C3b | ND | 7 | 0 | 49 | ND | <1 | 52 | 10 | ND |
| C3d | 15 | 0 | 5 | 14 | 1 | 0 | 33 | 5 | ND |
| Histology | Sezary cell leukaemia | T zone | Sezary cell | Sezary cell leukaemia | ML.HgU | T zone | — | ML.HgU | "T" PLL |

*Weak staining.
T zone = T zone lymphoma.
ML.HgU = Malignant lymphoma, high grade, unclassified.

lesion was clearly Leu-I$^+$, OKT3$^+$, OKT4$^+$ (mature T helper/inducer cell type), with few E$^+$ cells detected. Patient R.E. had 2 lymph node biopsies, at diagnosis and at relapse (R.E.2) after treatment. A pleural effusion (R.E.eff) was believed on cytological grounds to show malignant involvement. The phenotypic features in this case do not indicate dominance of a single subset of cells clearly showing two T cell markers. In both node biopsies the greater population is of null cell type (non-B non-T).

All populations in patient R.E. expressed HLA-ABC and were 2DI$^+$, suggesting a lymphoid or haematopoetic origin for the neoplastic cells, and in both node biopsies, OKT9 staining was detectable on the entire lymphoid population. Although the significance of this finding is obscure, it might indicate similarity with previously described cases where T cell markers were expressed (Kung *et al.*, 1981; Aisenberg & Wilkes, 1980).

### *Lymphoblastic lymphoma of T cell or null cell type*

The phenotype of ALL shows 3 major subclasses of disease: T cell type, C-ALL type, and B cell type. These sub-groups correspond histologically to the lymphoblastic lymphomas of childhood, which on morphological and cytochemical criteria can be segregated into T lymphoblastic lymphoma, unclassified lymphoblastic lymphoma (U) and lymphoblastic lymphoma of "Burkitt" type (B lymphoblastic lymphoma).

In the histological categories of T lymphoblastic and U lymphoblastic lymphoma, previous studies have indicated phenotypic similarities between the lymphoma and the corresponding cytological type of acute leukaemia; it is currently not clear whether the acute leukaemias are separate entities, or whether lymphoblastic lymphomas are variants of ALL (Habeshaw, 1981; Coccia, 1977; Murphy, 1978). In table XIII the phenotypic data are presented for one control thymus gland (C.W.), 6 cases of lymphoblastic lymphoma thought to be T cell type on histological and cytological criteria (patients J.P.C., K.F., A.N., S.B., J.P., J.C.) and 2 patients with lymphoblastic lymphoma of U cell type (patients L.T., Y.K.). The phenotype of the presenting lesion in patient L.T. (L.T.p) and of the recurrence after chemotherapy (L.T.r) are shown.

The control thymus gland (from a 22-year old female patient at thoracotomy) shows the majority of cells are E$^+$, αLeu-I$^+$, OKT3/UCHT-I$^+$, OKT4/αLeu-3a$^+$ and OKT8/α-2a$^+$. The marker OKT6 is also present on virtually all thymocytes. Reactivity with the monoclonal W6/32 shows the 43K glycoprotein core structure of HLA-AB(C) is not expressed by all thymocytes. HLA-DR is present on only a small population of intrathymic

**Table XIII** Phenotypes of involved tissue from patients with lymphoblastic lymphoma of T cell or unclassified type

| | *Patient Age/Sex Histology* | | | | | | | | | |
|---|---|---|---|---|---|---|---|---|---|---|
| | *JW* 22/*F* *Hyperplasia* (*Control*) | *JPL* 11/*M* *Skin* *TLB* | *JC* 13/*M* *Node* *TLB* | *AN* 7/*M* *Node* *TLB* | *KF* 3/*F* *Node* *TLB* | *JP* 14/*M* *Node* *TLB* | *SB* 16/*M* *Node* *TLB* | *LT* 7/*F* *Primary* *Node* *MLLB* (*U*) | *LT* 8/*F* *Recurrence* *MLLB* (*U*) | *YK* 11/*F* *Node* *MLLB* (*U*) |
| Markers | | | | | | | | | | |
| E | 96 | 66 | 12 | ND | 63 | 48 | 2 | 0 | 4 | 20 |
| Fcγ | ND | 4 | 1 | 0 | 3 | 0 | 1 | <1 | 0 | 1 |
| Fcμ | ND | 0 | <1 | 0 | <1 | 3 | 3 | 0 | 1 | 2 |
| C3b | ND | 0 | <1 | 0 | 0 | 1 | 0 | 0 | 0 | 0 |
| C3d | ND | 5 | 0 | 0 | 4 | 0 | 1 | 0 | <1 | 14 |
| SIg | 0 | 6 | 11 | 12 | 8 | 5 | 0 | 0 | 3 | 7 |
| αLeu-I | 83 | 58 | 89 | 30 | 80 | 60 | 100 | 65 | 30 | 24 |
| OKT3/UCHTI | 90 | 86 | 19 | ND | ND | 38 | <1 | ND | 9 | 20 |
| OKT4/antiLeu-3a | 90 | 14 | 20 | 80 | 7 | 64 | 97 | 1 | <1 | 20 |
| OKT8/antiLeu-2a | 81 | 72 | 12 | 70 | 50 | 95 | 2 | 2 | 2 | 6 |
| OKT9 | ND | 48 | 3 | ND | ND | 4 | 10 | ND | 28 | ND |
| OKT6 | 84 | 60 | 18 | 15 | 80 | 62 | 100 | 25 | 0 | 0 |
| J5 | 1 | 0 | 1 | <1 | 28 | 47 | 0 | 1 | 0 | ++* |
| PNA | 46 | 0 | 40 | 20 | 1 | ND | 0 | 0 | 0 | 3 |
| HLA ABC | 43 | 53 | 71 | 80 | 100 | 100 | 100 | 100 | 100 | 100 |
| HLA DR | 9 | 1 | 6 | 9 | 2 | 3 | 0 | <1 | 0 | +* |
| TdT | + | + | + | + | + | + | + | + | + | + |

Key: *Determination made on frozen section of affected node.
TLB Lymphoblastic lymphoma showing convoluted nuclei and/or acid phosphatase positivity.
Control: thymus removed at thoracotomy; histologically normal.
MLLB (U): Lymphoblastic lymphoma of cells without nuclear convolutions or acid phosphatase positivity.

cells, and the monoclonal J5 is not expressed on thymocytes. Almost half the thymocytes bind PNA, a finding confirmed by frozen section analysis. Thymocytes are also $TdT^+$.

In patient K.F., the predominant phenotype is $E^+$, $Leu\text{-}I^+$, $OKT8/Leu\text{-}3a^+$, $OKT6^+$, and $HLA^+$. (OKT4, $HLA\text{-}DR^-$ and $PNA^-$). Although in this case a $J5^+$ population was also present (28%) it appeared reciprocally related to the expression of OKT6. Patient A.N. showed predominant phenotype of $OKT4/Leu\text{-}3a^+$ and $OKT8/Leu\text{-}2a^+$ "double-marked" T cells, about half of which expressed Leu-I antigen. HLA-ABC was not present on 20% of cells, but it is of interest that the PNA binding and $OKT6^+$ population corresponds (proportionally) to the HLA-ABC negative T cell subset in this case. The 12% of $SIg^+$ B cells found were thought to represent residual lymph node cells, and are not considered part of the neoplasm. Patient S.B. showed a predominant population of $OKT6^+$, $Leu\text{-}I^+$, $OKT4/Leu\text{-}3a^+$ and $HLA\text{-}ABC^+$ T cells: ($E^-$, $OKT3/UCHT\text{-}I^-$, $OKT8/\alpha Leu\text{-}2a^-$ and $PNA^-$). Patient J.P. showed the presence of HLA-ABC, OKT6 and OKT8/Leu-2a antigens on virtually all cells. Only a subpopulation of cells were $OKT3/UCHT\text{-}I^+$, $OKT4/\alpha Leu\text{-}3a^+$ and $E^+$. This patient showed substantial numbers of $J5^+$ cells in the lesion, and the values obtained for J5 and αLeu-I indicate reciprocal expression of these 2 markers as in patient K.F. Patient J.P.L. showed a predominant $E^+$ $Leu\text{-}I^+$ $OKT3^+$ $OKT8^+$ population, which also expressed OKT6. Only half the cells expressed HLA-ABC. Peanut agglutinin staining was not seen. Patient J.C. showed a major $Leu\text{-}I^+$ population. A minority of cells were $OKT6^+$, and roughly the same proportion, $OKT3^+$, $OKT4^+$ and $OKT8^+$. HLA was present on only 71% of cells, and a significant proportion were also $PNA^+$.

These data indicate the existence of several populations of intrathymic, or "early" T cells, which do not correspond to T cell populations found in other lymphomas, or in normal tissues, outside the thymic microenvironment. Other markers (J5, W6/32, PNA) reveal further heterogeneity within apparently homogeneous T marked populations.

The patients L.T., Y.K. showed lesions which did not have the cytological features of T lymphoblastic lymphoma. In the first biopsy from patient L.T. (L.T.p) the majority of cells expressed αLeu-I antigen, with a small population expressing OKT6 (phenotype Leu-I$^+$ HLA$^+$, Leu-I$^+$, HLA$^+$ T6$^+$). In the second, post-relapse biopsy (L.T.r), no OKT6$^+$ cells were detected, the phenotype being essentially "null", with a minority of Leu-I$^+$ cells present. The lymph node in patient Y.K. showed a null profile on conventional marking. On frozen section the tumour cells were clearly J5$^+$, and HLA-DR$^+$. Most striking was the appearance, in the affected nodes, of islands of residual lymphocytes clearly negative for J5, but in which both mature T and B cells could be identified.

The intrathymic cell marker OKT6 is important for thymic cortical lymphocytes, with virtually no expression on lymphocytes outside the thymus, except in tissues involved with lymphoblastic lymphoma of T cell type. The marker J5 usefully identifies lymphoblast populations of C-ALL type, but this marker is also expressed in some centroblastic and centrocytic lymphomas, and can be positive on a subset of cells in T lymphoblastic lymphoma.

*Lymphoblastic lymphoma of B cell type*

Lymphoblastic lymphomas of B cell type are B cell-predominant, with few admixed T lymphocytes, and clear evidence of light chain class restriction. The phenotypic data presented for four such cases are shown in Table XIV. One of the cases (J.C.) expressed J5 antigen on the cells, and HLA-ABC was present in all cases. B cell status was confirmed by the concordance of HLA-DR$^+$ and SIg$^+$ cells in all cases. In patient L.A. where 54% of cells expressed only κ chain, the HLA-DR expressing population corresponded to the M κ SIg$^+$ population. In patient J.D., the E rosetting population exceeded the T cell population defined by the other T cell markers.

**Table XIV** Phenotype of involved tissue from patients with lymphoblastic lymphoma of B cell type (MLLB(B))

| *Patient Age/Sex Histology* | *JC 36/F MLLB* | *RW 4/M MLLB* | *JD 5/F MLLB* | *LA 2/M MLLB* |
|---|---|---|---|---|
| Markers | | | | |
| E | 4 | 3 | 48 | 6 |
| Fcγ | 1 | ND | 5 | 0 |
| Fcμ | 1 | ND | 24 | 0 |
| C3b | 0 | 0 | 8 | 0 |
| C3d | 8 | <1 | 12 | 0 |
| G | 12 | 0 | 84 | 2 |
| M | 75 | 69 | 14 | 36 |
| A | 14 | 0 | 10 | 0 |
| D | 6 | 0 | 16 | 0 |
| κ | 77 | 16 | 68 | 91 |
| λ | 2 | 78 | 5 | 0 |
| PNA | 10 | 4 | ND | ND |
| DA/2 | 20 | 77 | 46 | 72 |
| J5 | 54 | ND | 0 | ND |
| anti-LeuI | 14 | 3 | 28 | 8 |
| OKT3/UCHLTI | 15 | 3 | 16 | 10 |
| OKT4/αLeu-3a | 8 | 3 | 22 | 3 |
| OKT8/αLeu-2a | 6 | 4 | 6 | 11 |
| OKT9 | 14 | 10 | 0 | ND |
| OKT6 | 2 | 0 | 0 | ND |
| CyIg | Neg | Neg | Neg | Neg |
| T4/T8 ratio | 1.3 | 0.8 | 3.7 | 0.3 |

### Discussion

Monoclonal antibodies represent molecular probes which allow the detection of a single kind of antigenic structure upon the cell surface. The use of these reagents to classify cell subpopulations provides a practical means of defining the cellular composition of malignant lymphoma. The findings reported here, and previously published data on the specificity of monoclonal reagents (Reinherz *et al.*, 1979*a*, *b*; 1980 ) and on the distribution of the identified cell subsets in tissues (Janossy *et al.*, 1980; Poppema *et al.*, 1981) support the contention that T cell-specific monoclonal antibodies used in NHL consistently and reproducably define cell subsets which correspond closely to cells characterised functionally and phenotypically in normal tissues.

*T cell specific monoclonals*

The T cell specific monoclonals define several "families" of T cell associated antigens. These are:

1 OKT1/αLeu-I 65K MW glycoprotein antigen present on thymic and peripheral T cells, and shared by SIg$^+$ B cells in centroblastic and centrocytic lymphoma (patient P.A. (Table VIII)), centrocytic small cell lymphoma (patients P.C. and G.C. (Table IX)); immunoblastic lymphoma (patients J.B., J.N., F.B. (Table XII)). Leu-I$^+$ B cells do also occur as a normal lymphoid cell subset (Caligaris-Cappio *et al.*, 1982).
2 The OKT3/UCHT-I defined 19K MW glycoprotein antigen found mainly on peripheral T cell subsets, but which can occur separately from either Leu-I or the "helper/suppressor" subset antigens (see patients C.B. (Table VII), R.N. (Table VIII), G.S. and J.E. (Table X)).
3 The OKT4/αLeu-3a defined antigen found on T cell subsets with helper/inducer activity which occurs reciprocally with a second antigen

OKT8/Leu-2a expressed by T cell subsets with cytotoxic activity. Occasionally both antigens may appear together on the same cell (patient A.S. (Table XII)), particularly in thymus gland (J.W. (Table XIV)) or in T lymphoblastic lymphoma (Patient A.N. (Table XIV)).

4 The OKT8/αLeu-2a defined antigen confined to cytotoxic/suppressor T cell populations, which are found in thymus or T lymphoblastic lymphoma, or as a constituent of reactive nodes. No peripheral T cell malignancy of suppressor/cytotoxic subclass was found in this series, although reversed ratios of "helper/inducer:cytotoxic/suppressor" T cells were common in the described cases.

5 The receptor for sheep erythrocytes behaves as a separate T cell specific marker, and is quite often not represented on defined T cell populations of either helper/inducer or cytotoxic/suppressor subclasses (e.g. patients P.A., M.E., E.B. (Table VIII)). The antigen detected by OKT11 or OKT11a monoclonals shows similar distribution to the SRBC "receptor" (Verbi *et al.*, 1982).

6 The thymic cortical antigen defined by OKT6, present only in thymus gland or T lymphoblastic lymphoma. Although never found on lymphoid populations in reactive tissues, OKT6 expression can occur on Langerhans cells in skin, and in the sinuses of peripheral lymph nodes.

Since direct-coupled (FITC-or TRITC-labelled) monoclonals were not available for this study it was not practicable to test the simultaneous expression of 2 different antigens on one cell, therefore the existence of further heterogeneity of T cells in lymphoma beyond that described here cannot be excluded.

*Other monoclonal reagents*

*W6*/32 The monoclonal antibodies W6/32 (anti HLA-ABC) and DA-2 (anti HLA-DR) have appreciable value for phenotyping in suspension. W6/32 does not react with red cells, but reacts with all nucleated cells, including plasma cells, monocytes/macrophages, and epithelial cells. In thymus, W6/32 negative cells occur, and the presence of these cells bears an important relationship to the expression of αLeu-I defined antigen, OKT6-defined antigen and expression of PNA binding.

*DA*-2 The monoclonal DA-2 against HLA-DR is expressed by most B cells (but not by plasma cells) and in frozen section is also found to be strongly expressed by the inter-digitating reticulum cell of the T cell areas. Other HLA-DR positive cell classes include C-ALL antigen positive cells and blast transformed T4/Leu-3a$^+$ T cells (Janossy *et al.*, 1977; Reinherz *et al.*, 1979*c*).

*J5* The J5 monoclonal, which detects the 95K C-ALL antigen, is found to bind to some B cells in lymphomas, particularly of the follicular CB/cc subclass in suspension. J5 also reacted with a cell sub-population in OKT6$^+$ TdT$^+$ T lymphoblastic lymphoma. This C-ALL antigen expression at low levels in T lymphoblastic lymphoma has previously been noted using rabbit anti-ALL antibody (Habeshaw, 1981) and is present in some T lymphoblastic cell lines (Minowada *et al.*, 1978; Roberts *et al.*, 1978; Greaves *et al.*, 1981).

*2DI* The monoclonal antibody 2DI has proved very useful in eliminating from the series "receptor silent" tumours of uncertain histogenesis. All lymphoid cell populations presented here were defined by 2DI positivity confirming the validity of this marker for "lymphoid" populations in general.

*OKT9 expression in relation to histological class of lymphoma*

The monoclonal OKT9 did not act as a T lymphocyte specific marker. On the other hand, OKT9 expression was found to correlate with histological class of lymphoma as shown in Table XV.

It may be that the levels of OKT9 positivity reflect the proliferative potential within any class of lymphoma, since as shown here, the high grade lymphomas tend to show higher levels of OKT9$^+$ cells on average than low grade lymphoma (high grade $>10$, low grade $<5$). Since OKT9 detects the trf receptor, then rapidly growing cells, with a high metabolic demand for iron, may express receptors for trf in proportion to their rate of proliferation (Sutherland *et al.*, 1981). It is of further interest that a proportion of transforming B cell tumours express increased OKT 9 positivity, while T cell predominant transformation is not accompanied by increased OKT9 positivity.

*Phenotypic heterogeneity of null cells*

All the null cell populations described express HLA-ABC antigen and are found to be positive for HLeI by the monoclonal 2DI. "Null" cell populations occur frequently in lymphomas, and in reactive lymph nodes. The cell type marking as "null" has the phenotype HLA$^+$ 2DI$^+$ HLA-DR$^-$, and does not appear to express Fcγ, Fcμ, C3b or C3d receptors. Lymphomas having significant null populations of this type are centroblastic centrocytic follicular (Patients A.S., P.A., R.N. (Table VIII)), lymphocytic lymphoma (Patients E.H.

**Table XV** Correlation of OKT9-positive cells in lymphoma suspensions with histological grade of malignancy (excluding phenotypically-defined adult T cell lymphoma)

| *Histological high grade* | | *Histological low grade* | |
|---|---|---|---|
| *OKT9⁺ cells* | *Histology* | *OKT9⁺ cells* | *Histology* |
| 14 | LB(B) | 0 | MLL |
| 10 | LB(B) | 2 | MLL |
| 0 | LB(B) | 0 | MLL |
| 40 | LB(T) | 0 | MLL |
| 3 | LB(T) | 0 | MLL |
| 4 | LB(T) | 1 | MLL |
| 10 | LB(T) | 0 | MLL |
| 28 | LB(U) | 2 | MLL |
| 58 | MLIB | 2 | MLL |
| 18 | MLIB | 4 | MLL |
| 2 | MLIB | 0 | MLL |
| 19 | MLIB | 22 | ML/cc/SC |
| 5 | MLIB | 0 | ML/cc/SC |
| 6 | MLIB | | |
| 51 | MLIB | 4 | ML/cc/LC |
| 42 | MLIB | 1 | ML/cc/LC |
| 11 | MLIB | 0 | ML/cc/LC |
| 5 | MLIB | 0 | ML/cc/SC |
| 4 | CB/cc/F (T) | 0 | ML/cc/SC |
| 60 | CB/cc/F (T) | 2 | CB/cc/F |
| 0 | CB/cc/F (T) | 1 | CB/cc/F |
| 18 | MLCB(P) | 0 | CB/cc/F |
| 22 | MLCB | 2 | CB/cc/F |
| 8 | MLCB | 1 | CB/cc/F |
| 6 | MLCB | 3 | CB/cc/F |
| 7 | MLCB(P) | 5 | CB/cc/F |
| | | 2 | CB/cc/F |
| | | 4 | CB/cc/F |
| | | 0 | CB/cc/F |
| 18 | Mean value | 2 | Mean value |
| (0–60) | Range | (0–22) | Range |
| 26 cases | | 28 cases | |

and H.M. (Table XII)) and "T zone" lymphoma (patient R.E. (Table XIII)). Null populations were not found in centrocytic lymphoma, centroblastic lymphoma, or immunoblastic lymphoma. Previous reports of "null" populations include $E^-$ T cells and Leu-$I^+$ $SIg^-$ HLA-$DR^+$cells, which can only be sub-classified using specific monoclonals. Failure to find CyIg, the absence of HLA-DR, and the apparent lack (or inconsistent expression) of $Fc\gamma$ and C3b receptors suggest the null cell is neither a plasma cell, nor a mononuclear phagocyte of conventional type. It is thought unlikely to be of B cell lineage, but could represent a subset of T lineage cells, not detectable with current markers. This is suggested from the existence of populations bearing only the T cell specific marker.

In addition to the HLA-$ABC^+$ $2DI^+$ HLA-$DR^-$ "null" cell, other cells are found which express only one class specific marker (other than SIg), These include the $E^+$ cell with no expression of other T cell markers ($HLA^+$ $2DI^+$ $E^+$) and (Leu-$I^+$ HLA-$DR^+$) phenotype. The former phenotype occurred in immunoblastic lymphoma (J.P. (Table X)), centroblastic lymphoma (M.B. (Table VII)) and lymphoblastic lymphoma (J.D. (Table XV)). Leu-$I^+$ cells of the phenotypes (HLA-$ABC^+$ $2DI^+$ Leu-$I^+$ $SIg^+$ and/or HLA-$DR^+$) were present in centroblastic and centrocytic follicular lymphoma (P.A. (Table VIII)), centrocytic lymphoma of small cell type (patients P.C., J.E., J.M. (Table X)) and lymphocytic lymphoma (Patients J.B., J.N. and F.B. (Table XII)).

*Phenotypic heterogeneity in peripheral T cells*

There is evidence from this series that the antigens detected by the monoclonals αLeu-I (OKT1), OKT6, OKT3/UCHT-I, OKT4/αLeu-3a or OKT8/αLeu-2a, and E rosette formation provide a number of separate T cell phenotypes dependent upon the independent expression of these antigens. Thus, although, peripheral T cells are normally $E^+$, OKT3/UCHT-I$^+$, OKT4/Leu-3a$^+$ or OKT8/Leu-2a$^+$, independent expression of these markers does occur, giving rise to $E^-$, OKT3/UCHT-I$^+$, OKT4/Leu-3a$^-$, OKT8/Leu-2a$^-$ phenotypes within the cell series classified as "T". It was found that the T subset specific markers OKT4/αLeu-3a, OKT8/αLeu-2a never occurred without expression of OKT3/UCHT-I and/or Leu-I. There is no evidence that expression of OKT3/UCHT-I antigen or Leu-I antigen is dependent on OKT4/Leu-3a, OKT8/Leu-2a antigen expression. $E^-$ T cell populations are common ($E^-$ OKT3/UCHT-I$^+$), occurring with both OKT4/Leu-3a positive and OKT8/Leu-2a$^+$ subclass specific markers. The monoclonal antibody OKT11a, which detects the sheep erythrocyte receptor (Verbi *et al.*, 1982) was not used in this series; the results obtained with E rosetting would also apply to this antibody. $E^-$ T cell populations were found in centroblastic lymphoma (patients I.C., J.S., C.B. (Table VII)), in centroblastic/centrocytic follicular lymphoma (patients P.A., D.C., M.E., E.B. (Table VIII)) and in patients with T cell lymphoma (E.M. (blood) and P.J. (Table XIII)). In a number of other cases E rosette levels were somewhat below total T cell levels determined by pan T cell markers (e.g. patient D.L. (Table IX)), probably due to the inherent variability of the rosette technique.

In 4 cases (Patient C.B., (Table 7), R.N. (Table VIII), G.S. and J.E. (Table X)) a significant proportion of the total T cells expressed the pan T cell markers αLeu-I and/or OKT3/UCHT-I only. In these patients total T "helper" and "suppressor" T cells did not equal total T cells. The differences encountered were not great, but do suggest the presence of an undefined peripheral T cell with phenotype Leu-I$^+$ OKT3/UCHT-I$^+$ ($E^+$), unreactive with OKT4/αLeu-3a or OKT8/αLeu-2a antibodies.

"Double marked" OKT4$^+$ OKT8$^+$ T cell populations have been described (e.g. in prolymphocytic leukaemia) and also occur in thymus gland (Greaves *et al.*, 1981). In this series, only one case (A.B. (CB/cc/F—Table VIII)) showed clearly a double-marked T cell population.

*Phenotypic heterogeneity of intrathymic T cell subsets*

It is believed that the phenotypes described of T cell precursors and T cells of normal thymus gland, are analogous to those of lymphoblastic leukaemia of T cell type (Bhan *et al.*, 1980; Greaves *et al.*, 1981).

In lymphoblastic lymphoma, homogeneous populations can be found (e.g. patient S.B. (Table XIV) Leu-I$^+$, OKT6$^+$, OKT4$^+$, HLA$^+$, PNL$^-$) but in other cases the tumour cell populations all represent mixtures of cells of different phenotypes with variable levels of expression of the T cell markers. The markers αLeu-I OKT6, W6/32 (HLA-ABC), J5 and PNA are of further interest in this class of lymphoma, since there appears to be reciprocal expression of some phenotypic features (e.g. HLA-ABC and PNA) in normal thymus gland and in lymphoblastic lymphoma. In patient L.T.p, the neoplasm contains apparently two populations: Leu-I$^+$, HLA-ABC$^+$, OKT6$^-$ cells (65%) and Leu-I$^-$, HLA-ABC$^+$, OKT6$^+$ cells (25%). In patient A.N., a presumptive population of Leu-I$^+$ OKT6$^+$ and PNA$^+$ (15–30%) cells was HLA-ABC$^-$. In patient K.F., Leu-I$^+$ OKT6$^+$ HLA$^+$ cells accompanied a J5$^+$, HLA-ABC$^+$ HLA-DR$^-$ population. These data suggest that there is an important relationship between the antigens defined by αLeu-I, OKT6, HLA-ABC and J5 in intrathymic differentiation.

The experience with monoclonal antibodies against defined T cell subsets in T ALL and in T LBL illustrates clearly that several variations in expression of related antigens (e.g. HLA-ABC and OKT6 defined antigens) are possible within the thymic microenvironment, and that lymphoblastic lymphoma phenotypes are illustrative examples of some of these variations. The total range of phenotype within these classes of malignancy obviously may be very great without necessarily rejecting the hypothesis that the phenotype expressed on the neoplastic population is equivalent to the phenotype expressed on normal cells at an equivalent stage of differentiation of maturation. In particular, essential evidence of the normal phenotype of intrathymic T cells in thymuses of different chronological age is not available, although it is known that "foetal" and "mature" thymuses show different phenotypes.

In a recent paper, Bernard *et al.* (1981) reported a series of 21 patients with lymphoblastic lymphoma, and found phenotypic variants similar to those described here. Commonly T lymphoblastic lymphoma cells expressed T6 antigen with both OKT4 and OKT8 expressed, together with the thymocyte specific monoclonal OKT10. Three patients in their series expressed J5 reactivity, and 2 patients expressed both OKT3 and OKT6 antigens. The monoclonal A50 (Boumsell *et al.*, 1980) which recognises an antigen present on peripheral T cells, but absent from the majority of thymocytes showed reciprocal expression in T all (A50$^+$, 1/18 cases) and lymphoblastic lymphoma (A50$^+$, 10/21 cases). Variations in expression of the HLA-ABC antigens and PNA binding status were not reported. These authors present evidence showing phenotypic differences between lymphoblastic lymphoma and T-ALL, and imply that lymphoblastic lymphoma cells are later (i.e. more mature) variants of early T cells than T-ALL cells.

*Phenotypic heterogeneity of B cell subsets*

In the absence of data for the available monoclonal markers of B lymphocyte subsets, the most valid marker for B cell subset heterogeneity remains the expressed heavy chain isotype. The restriction of B cells with expressed $\mu+\delta$ heavy chains (HC) to the lymphocyte mantle or corona around germinal centres is very striking, particularly in view of the preservation of this distribution in follicular lymphoma containing B cells of this phenotype (Figure Ie). Moreover, lymphomas of B cells can express either single or multiple HC isotypes on the neoplastic population. In terms of HC class expression, lymphomas of lymphoblastic B cell type and centroblastic type are frequently of single IgM HC class, while multiple HC isotype expression is more frequent in follicular lymphomas. Lymphomas of more "mature" B cells tend to express single H chain isotype of IgG or IgA class. There is no clearcut HC isotype relationship to histological class.

A subset of B cells also expresses the antigen defined by the αLeu-I monoclonal in 3 classes of lymphoma: follicular, immunoblastic and lymphocytic. Recent evidence that αLeu-I-like antibody (RFA-I) identifies a mouse red blood cell-positive SIg$^+$ B cell subset in normal tonsil (equivalent in phenotype to the CLL cell) implies that a Leu-I bearing B cell may be a significant component of B CLL, CB/cc/F lymphoma, and some cases of immunoblastic lymphoma. This is of interest, since a postulated bone marrow precursor cell of the germinal centre B cell has not so far been formally identified. If Leu-I$^+$ B cells are (as suggested by Caligaris-Cappio *et al.*, 1982) of peripheral origin and represent early "B memory" cells, the germinal centre B cell precursor may well prove to be a C-ALL$^+$ SIg$^+$ and HLA-DR$^+$ B lineage cell—a significant B cell component of most follicular lymphomas. The presence of C-ALL antigen has been previously shown on some cases of B lymphoblastic lymphoma (Habeshaw, 1981), and pre-B cells in C-ALL, and would suggest that the lymphoblastic lymphoma of B cell type in children, and CB/cc/F lymphoma in adults, may be clearly related in their ontogenesis, despite the contrasting histological appearances and clinical behaviour.

With the means to describe accurately the T cell populations in NHL, there is a temptation to regard deviations of T cell phenotype from the prescribed "T helper/inducer" or "T cytotoxic/suppressor" subsets as evidence of "neoplastic" transformation of T cells with or without B cells in lymphoma. In our opinion there is no evidence that unusual phenotypes (such as OKT4/OKT8 double-marking T cell populations) are necessarily "neoplastic" attributes of lymphoid cells. Nor is there evidence from the phenotype to suggest that proliferative B cell malignancies are caused by unopposed "T helper" cell function or are subject to ineffective "T suppression", as both types of cell are usually present in normal proportions. In normal or reactive nodes, the T cell component is of both major subclasses, is arranged in a similar distribution, and presumably has the same function as the T cell component in, for example, centroblastic and centrocytic follicular lymphoma. The excess of OKT8/Leu-2a$^+$ T cells over OKT4/Leu-3a$^+$ T cells commonly observed in this series is also observed, though less commonly, in reactive lymph nodes, but does not alone imply a reaction by "cytotoxic" T cells against "neoplastic" B cells. Moreover, there is good evidence that the clonogenic components of NHL B cells are, like normal B cells, dependent upon T cell factors, and T cells, for growth (Izaguirre *et al.*, 1980).

If abnormal (or "neoplastic") T cell phenotypes are to be found, they would presumably be most common in T cell lymphomas. Again, where this is observed, T cell lymphomas appear to have quite clearly developed attributes of T "helper" cells (Kung *et al.*, 1981), or phenotypes compatible with origins from within thymus gland (Bernard *et al.*, 1981).

However, a proportion of high grade lymphomas, histologically and cytologically defined as being of "T cell" type, include light chain class restricted B lymphoid components expressing surface and cytoplasmic Ig. Similarly, B cell immunoblastic lymphoma, of restricted L chain class, is often accompanied by a major population of active, and in some cases blast transformed HLA-DR$^+$ T lymphocytes in the lesion.

In this respect, previously published data (Habeshaw *et al.*, 1979) indicate that repeat biopsies of T cell predominant lesions show T cell predominance to be merely one phase in the evolution of a dominant light chain class-restricted B cell population with apparent malignant potential. For understanding the nature of lymphoma it is important to establish whether the T cell predominance is associated with the normal immunoregulatory mechanisms of T cells upon B cells rather than concluding from the local predominance of one cell class that the neoplasm was essentially composed of "malignant" cells of that type. For this reason the phenotypic criteria adopted for defining a neoplasm as "T cell type" are stringent, and emphasise the necessity of looking for and excluding significant "monoclonal" B cell populations in the affected tissues.

Suspension phenotyping shows that most NHL are composed of mixtures of cells. These mixtures include T cells of both major subsets, and B cells which may show heterogeneity of heavy chain isotype, HLA-DR expression, markers such as C3b/C3d receptor expression, or PNA binding. Even T lymphoblastic lymphomas show some evidence of being composed of mixtures of phenotypically distinct subsets of cells, which may mimic the normal patterns of phenotypic variation occurring during intrathymic differentiation. The T cell component in Hodgkin's Disease (Dorreen et al., 1982) or in follicular lymphoma is often not demonstrably abnormal in respect of the relative proportions of T "helper" or "suppressor" cells, or in their distribution as assessed by frozen section. Nonetheless, mixtures of such cells do, in individual patients, show the capacity for progressive growth, ultimately with the emergence of a dominant B cell population showing light chain class restriction, and probably derived from a single B cell clone. From these observations, it is proposed that T cell populations in NHL of "B" cell type are an integral part of the proliferative process, acting in the selection and stimulation of the B cell component in a manner analogous to the normal immune response. The presence of a progressive, and ultimately fatal, lymphoid proliferation represents a rather gross disturbance of normal function which is not always reflected in the cellular composition of the neoplasm itself. This observation suggests that the presence of a lymphoid neoplasm may be symptomatic of a more widespread constitutional disturbance of lymphoid homeostasis affecting the immune system as a whole, rather than being the expression of an intrinsic "malignant" attribute of a single B cell clone. In view of the reported recent success in the treatment of a lymphoma by monoclonal anti-idiotypic antibody (Miller et al., 1982), defective immunoregulation at this level might well prove to be a major factor in the pathogenesis of lymphoid neoplasia.

The authors would like to acknowledge the clinical co-operation of Dr T.A. Lister and Professor J.S. Malpas of the Medical Oncology Unit, St. Bartholomew's Hospital. We should also like to thank Dr S. Schlossman, Dr J. Ritz, Dr A. McMichael, Dr G. Goldstein, Dr P. Beverley and Dr W. Bodmer for their kind gifts of monoclonal reagents. The technical assistance of Margaret Rainey and Henrietta Taylor is acknowledged. The manuscript was typed by Mrs J. Barton.

### Appendix

*Monoclonal antibodies used in this study*

Additional references are cited in some cases to monoclonal reagents of apparently similar specificity.

*αLeu-1* Antibody defining glycoprotein 69-71K antigen (P69–71) on normal T lymphocytes in peripheral lymphoid tissue, a normal B cell subset (Janossy personal communication), and $SIg^+$ B cells of CLL type (Wang et al., 1980). Similar antibodies are clone P17 F12, IgG2a antibody identifying a 67K MW antigen of similar distribution (Engleman et al., 1981), T65 reacting with a 65K MW antigen (Royston et al., 1980) and clone A50, IgG2a antibody, reactive with a T cell subset and CLL cells (Boumsell et al., 1980).

*αLeu-2a* Clone SKI, an IgG antibody defining an antigen complex of 2 or more disulphide bonded subunits of 32K and 43K MW. Present on a subset of T lymphocytes responsible for cell-mediated lymphosis (suppress or/cytotoxic subset) (Ledbetter et al., 1981).

*αLeu-3a* Clone SK3, an IgGI antibody defining a 55K MW antigen present upon T cells mediating PWM-induced B cell antibody synthesis (Evans et al., 1981).

*OKT1* An IgGI antibody reacting with 70K MW antigen expressed on all peripheral T cells and a subpopulation of thymocytes (Reinherz et al., 1979 ). P17/F12 and T101 react with the same antigen (Engleman & Levy, 1980).

*OKT3* Monoclonal IgG2a antibody reacting with a 19K MW antigen expressed on all peripheral T cells, including both suppressor/cytotoxic and helper/inducer subclasses (Kung et al., 1979).

*OKT4* Monoclonal IgG2b antibody reactive with a 62K MW antigen present on human T cells of helper/inducer subclass ($TH_2$—T cells) (Kung et al., 1979; Reinherz et al., 1979a,b).

*OKT6* Monoclonal IgG antibody reactive with a 49K MW antigen expressed only on thymocytes, and absent from peripheral T cells (Reinherz et al., 1980a). Similar monoclonal is Nal/34 (McMichael et al., 1979).

*OKT8* Monoclonal IgG2a antibody reactive with an undefined antigen of peripheral T cells of cytotoxic/suppressor subclass ($TH_2^+$) (Reinherz et al., 1980b). A similar but not identical antibody is OKT5 (Reinherz et al., 1980b).

*W6/32* Monoclonal IgG2a antibody reactive with the 43K glycoprotein "core" chain of HLA A, B, (C) antigens (Parnham et al., 1979).

26/114 Monoclonal IgG2a antibody reactive with the 12K MW protein $\beta_2$ M (Trucco et al., 1979).

*DA*-2 Monoclonal IgG2a antibody directed against polymorphic p28/33 glycoprotein "core" structure of HLA-DR determinants (Brodsky *et al.*, 1979).

*2DI* Monoclonal IgGI antibody directed against a human haematopoietic cell antigen (HLeI) of 70K MW, present on lymphoid and myeloid cells, weakly expressed on granulocytes, monocytes and early erythroid precursors. Absent from a wide variety of tested epithelia (Beverly *et al.*, 1980).

*J5* Monoclonal mouse IgG2a antibody reactive with the 95K antigen of C-ALL cells (Ritz *et al.*, 1980).

*AN*51 Monoclonal antibody reactive with human platelet glycoprotein I. Unreactive with human lymphoid cells (McMichael *et al.*, 1981).

*OKT*9 Monoclonal antibody reactive with an 80K dimeric antigen expressed on thymic T cell (Reinherz *et al.*, 1980*a*). It is now known to be reactive with the cellular receptor for trf (Sutherland *et al.*, 1981) with widespread cellular distribution.

*UCHT-I* Monoclonal antibody reactive with 19K MW antigen, expressed on peripheral T cells showing identical reactivity to the monoclonal OKT3 (Beverley P, personal communication).

**References**

AISENBERG, A.C. & WILKES, B.M. (1980). Unusual lymphoma phenotype defined by monoclonal antibody. *J. Exp. Med.*, **152,** 1126.

AISENBERG, A.C. (1981). Current concepts in immunology. Cell surface markers in lymphoproliferative disease. *N. Engl. J. Med.*, **304,** 331.

BERNARD, A.B., BOUMSELL, L., REINHERZ, E.L. & 9 others. (1981). Cell surface characteristics of malignant T cells from lymphoblastic lymphoma using monoclonal antibodies. Evidence for phenotypic differences between malignant T cells from patients with acute lymphoblastic leukaemia and pymphoblastic lymphoma. *Blood,* **57,** 1105.

BEVERLY, P., LINCH, D. & DELIA, D. (1980). Isolation of human haematopoetic progenitor cells using monoclonal antibodies. *Nature,* **287,** 332.

BHAN, A.K., REINHERZ, E.L., POPPEMA, S., MCCLUSKEY, R.T. & SCHLOSSMAN, S.F. (1980). Location of T cell and major histocompatibility complex antigens in the human thymus. *J. Exp. Med.* **152,** 771.

BLOOMFIELD, C.D., GAJL-PECZALSKA, K., FRIZZERA, G., KERSEY, J.H. & GOLDMAN, A.I. (1979). Clinical utility lymphocyte surface markers combined with the Lukes-Collins histological classification in adult lymphoma. *N. Engl. J. Med.*, **301,** 512.

BOUMSELL, L., COPPIN, H., PHAM, D. & 4 others. (1980). An antigen shared by human T cell subset and B cell chronic lymphocytic leukaemia cells. *J. Exp. Med.*, **152,** 229.

BRODSKY, F.M., PARNHAM, P., BARNSTABLE, C.J., CRUMPTON, M.J. & BODMER, W.F. (1979). Hybrid myeloma monoclonal antibodies against MHC products. *Immunol. Rev.*, **47,** 3.

BROUET, J.C., PREUD'HOMME, J.L. & SELIGMANN, M. (1976). Lymphocyte membrane markers in B cell proliferations and human non-Hodgkin's lymphomas. In: *Clinical Tumour Immunology* (Eds. Wybran & Staquet) Oxford: Pergamon Press p. 123.

CALIGARIS-CAPPIO, F., GOBBI, M., BOFIL, M. & JANOSSY, G. (1982). Infrequent normal B lymphocytes express features of B-chronic lymphocytic leukaemia. *J. Exp. Med.*, **155,** 623.

COCCIA, P.F. (1977). Characterisation of the blast cells in childhood non-Hodgkins lymphoproliferative malignancies. *Semin. Oncol.*, **4,** 287.

DORREEN, M.S., HABESHAW, J.A., WRIGLEY, P.F.M. & LISTER, T.A. (1982). Distribution of T-lymphocyte subsets in Hodgkin's disease characterised by monoclonal antibodies. *Br. J. Cancer,* **45,** 491.

ENGLEMAN, E.G. & LEVY, R. (1980). Immunologic studies of a human T lymphocyte antigen recognised by a monoclonal antibody. *Clin. Res.*, **28,** 511A.

ENGLEMAN, E.G., WARNKE, R., FOX, R.I. & LEVY, R. (1981). Studies on a human T lymphocyte antigen recognised by a monoclonal antibody. *Proc. Natl Acad. Sci.*, **58,** 1791.

EVANS, R.L., LAZARUS, H., PENTA, A.C. & SCHLOSSMAN, S.F. (1978). Two functionally distinct subpopulations of human T cells that collaborate in the generation of cytotoxic cells responsible for cell-mediated lympholysis. *J. Immunol.* **129,** 1423.

EVANS, R.L., WALL, D.W., PLATSOUCAS, C.D. & 4 others. (1981). Thymus dependent membrane antigens in man: Inhibition of cell-mediated lympholysis by monoclonal antibodies to the TH-2 antigen. *Proc. Natl Acad. Sci.*, **78,** 544.

FORNI, L. (1979). Reagents for immunofluorescence and their use for staining lymphoid cell products. In: *Immunological Methods* (Eds. Lefkouits & Pernis), New York: Academic Press p. 151.

GAJL-PECZALSKA, K.J., BLOOMFIELD, C.D., COCCIA, P.F., SOSIN, H., BRUNNING, R.D. & KERSEY, J.H. (1975). B and T cell lymphomas. Analysis of blood and lymph nodes in 87 patients. *Am. J. Med.*, **59,** 674.

GREAVES, M.F., RAO, J., HARIRI, G. & 4 others. (1981). Phenotypic heterogeneity and cellular origins of T cell malignancies. *Leukaemia Res.*, **5,** 281.

HABESHAW, J.A., MACAULAY, R.A.A. & STUART, A.E. (1977). Correlation of surface receptors with histological appearance in 29 cases of non-Hodgkin lymphoma. *Br. J. Cancer,* **40,** 858.

HABESHAW, J.A., CATLEY, p., STANSFELD, A.G. & BREARLEY, R.L. (1979). Surface phenotyping, histology, and the nature of non-Hodgkin lymphoma in 157 patients. *Br. J. Cancer,* **40,** 20.

HABESHAW, J.A. (1981). Heterogeneity of lymphoblastic malignancies in children. *J. Cancer Res. Clin. Oncol.*, **101**, 23.

IZAGUIRRE, C.A. & HABESHAW, J.A. (1982). Abnormal differentiation of malignant B cells in non-Hodgkin lymphoma. *J. Cell Biochem.* (*suppl.*) **6**, 10 (abstract 0042).

IZAGUIRRE, C.A., MINDEN, M.D., HOWATSON, A.F. & MCCULLOCH, E.A. (1980). Colony formation by normal malignant human B lymphocytes. *Br. J. Cancer*, **42**, 430.

JAFFE, E.S., SHEVACH, E.M., FRANK, M.M., BERARD, C.W. & GREEN, I. (1974). Nodular lymphoma, evidence for an origin from follicular B lymphocytes. *N. Engl. J. Med.*, **290**, 813.

JAFFE, E.S., BRAYLAN, R.C., NANBA, K., FRANK, M.M. & BERARD, C.W. (1977). Functional markers, a new perspective on malignant lymphomas. *Cancer Treat. Rep.*, **61**, 953.

JANOSSY, G., GOLDSTONE, A.H., CAPELLARO, D. & 4 others. (1977). Differentiation-linked expression of p. 28, 33 (Ia-like) structures on human leukaemic cells. *Br. J. Haematol.*, **37**, 391.

JANOSSY, G., TIDMAN, N., SELBY, W.S., THOMAS, J.A., GRANGER, S., KUNG, P.C. & GOLDSTEIN, G. (1980). Human inducer and suppressor T lymphocytes occupy different microenvironments. *Nature*, **288**, 81.

JANOSSY, G., THOMAS, J.A., PIZZOLO, G. GRANGER, S.M., MCLAUGHLIN, J., HABESHAW, J.A., STANSFELD, A.G. SLOAN, J. (1980). Immuno-histological diagnosis of lymphoproliferative diseases by selected combinations of antisera and monoclonal antibodies. *Br. J. Cancer*, **42**, 2244.

KUNG, P.C., GOLDSTEIN, G., REINHERZ, E.L. & SCHLOSSMAN, S.F. (1979). Monoclonal antibodies defining distinctive human T cell surface antigens. *Science*, **206**, 347.

KUNG, P.C., BERGER, C.L., GOLDSTEIN, G., LOGERFO, P., EDELSON, R.L. (1981). Cutaneous T cell lymphoma: Characterisation by monoclonal antibodies. *Blood*, **57**, 261.

LEDBETTER, J.A., EVANS, R.L., LIPINSKY, M., CUNNINGHAM, RUNDLES, C., GOOD, R.A. & HERZENBERG, L.A. (1981). Evolutionary conservation of surface molecules that distinguished T lymphocyte helper/inducer and T cytotoxic/suppressor subpopulations in mouse and man. *J. Exp. Med.*, **153**, 310.

LEVY, R., DILLEY, J., FOX, R.I. & WARNKE, R. (1979). A human thymus-leukaemia antigen defined by hybridoma monoclonal antibodies. *Proc. Natl Acad. Sci.*, **76**, 6552.

LUKES, R.K., TAYLOR, C.R., PARKER, J.W., LINCOLN, T.L., PATTENGALE, P.K. & TINDLE, B.H. (1978). A morphologic and immunologic surface marker study of 299 cases of non Hodgkin's and related leukaemias. *Am. J. Pathol.*, **90**, 461.

MCMICHAEL, A.J., PILCH, J.R., GALFRE, G., MASON, D.Y., FABRE, J.W. & MILSTEIN, C. (1979). A human thymocyte antigen defined by a hybridoma monoclonal antibody. *Eur. J. Immunol.*, **9**, 205.

MCMICHAEL, A.J., RUST, N.A., PILCH, J.R. & 6 others. (1981). Monoclonal antibody to human platelet glycoprotein I. Immunological studies. *Br. J. Haematol.*, **49**, 501.

MILLER, R.A., MALONEY, D.G., WARNKE, R. & LEVY, R. (1982). Treatment of a B cell lymphoma with monoclonal anti-idiotype antibody. *N. Engl. J. Med.*, **306**, 517.

MINOWADA, J., JANOSSY, G., GREAVES, M.F., TSUBOTA, T. & 3 others. (1978). Expression of an antigen associated with acute lymphoblastic leukaemia in human leukaemia-lymphoma cell lines. *J. Natl Inst.*, **60**, 1269.

MURPHY, S.B. (1978). Current concepts in cancer. Childhood non-Hodgkin lymphoma. *N. Engl. J. Med.*, **299**, 1446.

PARNHAM, P., BARNSTABLE, C.J. & BODMER, W.F. (1979). Properties of an anti-HLA ABC monoclonal antibody. Use of a monoclonal antibody W6/32 in structural studies of HLA ABC antigens. *J. Immunol.*, **123**, 342.

POPPEMA, S., BHAN, A.K., REINHERZ, E.L., MCCLUSKEY, R.T. & SCHLOSSMAN, S.F. (1981). Distribution of T cell subsets in human lymph nodes. *J. Exp. Med.*, **153**, 30.

REINHERZ, E.L., KUNG, P.C., GOLDSTEIN, G., LEVY, R.H. & SCHLOSSMAN, S.F. (1980*a*). Discrete stages of human intrathymic differentiation: Analysis of normal thymocyte and leukaemic lymphoblasts of T cell lineage. *Proc. Natl Acad. Sci.*, **77**, 1588.

REINHERZ, E.L., KUNG, P.C., GOLDSTEIN, G., SCHLOSSMAN, S.F. (1980*b*). A monoclonal antibody reactive with the human cytotoxic/suppressor T cell subset previously defined by a heteroantiserum termed TH2. *J. Immunol.*, **124**, 1301.

REINHERZ, E.L., KUNG, P.C., GOLDSTEIN, G. & SCHLOSSMAN, W.F. (1979*a*). A monoclonal antibody with selective reactivity for functionally mature human thymocytes and all peripheral human T cells. *J. Immunol.*, **123**, 1312.

REINHERZ, E.L., KUNG, P.C., GOLDSTEIN, G. & SCHLOSSMAN, S.F. (1979*b*). Further characterization of the human inducer T cell subset defined by monoclonal antibody. *J. Immunol.*, **123**, 2894.

REINHERZ, E.L., KUNG, P.C., GOLDSTEIN, G. & SCHLOSSMAN, S.F. (1979*c*). Separation of functional subsets of human T cells by a monoclonal antibody. *Proc. Natl Acad. Sci.*, **76**, 4061.

REINHERZ, E.L., KUNG, P.C., PESANDO, J.M., RITZ, J., GOLDSTEIN, G. & SCHLOSSMAN, S.F. (1979*d*). Ia determinants on human T cell subsets defined by monoclonal antibody: Activation stimuli required for expression. *J. Exp. Med.*, **150**, 1472.

RITZ, J., PESANDO, J.M., MCCONARTY, J.N., LAZARUS, H. & SCHLOSSMAN, S.F. (1980). A monoclonal antibody to human acute lymphoblastic leukaemia antigen. *Nature*, **283**, 583.

ROBERTS, M., GREAVES, M.F., JANOSSY, G., SUTHERLAND, R. & PAIN, C. (1978). Acute lymphoblastic leukaemia (ALL) associated antigen I. Expression in different haematopoetic malignancies. *Leukaemia Res.*, **2**, 105.

ROSE, M.L., BIRBECK, M.S.C., WALLIS, V.J., FORRESTER, J.A. & DAVIES, A.J.S. (1980). Peanut lectin binding properties of germinal centres of mouse lymphoid tissue. *Nature*, **284**, 364.

ROSE, M.L., HABESHAW, J.A., KENNEDY, R., SLOANE, J., WILTSHAW, E. & DAVIES, A.J.S. (1981). Binding of peanut lectin to germinal centre cells. A marker for B

cell subsets of follicular lymphoma. *Br. J. Cancer*, **44**, 68.

ROYSTON, I., MAJDA, J.A., BAIRD, S.M., MESERVE, B.L. & GRIFFITHS, J.C. (1980). Human T cell antigens defined by monoclonal antibodies. The 65,000 Dalton antigen of T cells (T65) is is also found on chronic lymphatic leukaemia cells bearing surface immunoglobulins. *J. Immunol.*, **125**, 725.

STATHOPOULOS, G., PAPMICHAIL, M., HOLBOROW, E.J. & DAVIES, A.J.S. (1977). Description of the cells from the lymph nodes of patients with non Hodgkin lymphoma according to some B and T cell characteristics. *Br. J. Exp. Pathol.*, **58**, 95.

STEIN, H. (1976). Immunochemische und immuzytologische Befunde bei non-Hodgkin lymphomen. *Haematol. Bluttransfus.*, **18**, 167.

STEIN, H., TOLKSDORF, G., BURKERT, M. & LENNERT, K. (1979). Cytological classification on non Hodgkin's lymphomas based on morphology, cytochemistry and immunology. *Ad. Med. Oncol. Res. Ed.*, **7**, 141.

STRAUCHEN, J.A., YOUNG, R.C., DeVITA, V.T., ANDERSON, T., FANTONE, T.C. & BERARD, C.W. (1978). Clinical relevance of the histopathological subclassification of diffuse "histiocytic" lymphoma. *N. Engl. J. Med.*, **299**, 1382.

SUTHERLAND, R., DELIA, D., SCHNEIDER, C., NEWMAN, R., KEMSHEAD, J. & GRAVES, M. (1981). Ubiquitous cell surface glycoprotein on tumour cells is proliferation associated receptor for transferrin. *Proc. Natl Acad. Sci.*, **78**, 4515.

TRUCCO, M.M., GAROTTA, G., STOKER, J.W. & CEPPELINI, R. (1979). Murine monoclonal antibodies against HLA structures. *Immunol. Rev.*, **47**, 287.

VERBI, W., GREAVES, M.F., SCHNEIDER, C., KOUBEK, K., JANOSSY, G., STEIN, H., KUNG, P. & GOLDSTEIN, G. (1982). Monoclonal antibodies OKT11 and OKT11a have pan T cell reactivity and block sheep erythrocyte receptors. *Eur. J. Immunol.*, **12**, 81.

WANG, C.Y., GOOD, R.A., AMMIRATI, P., DYMBORT, G. & EVANS, R.L. (1980). Identification of a p69 71 complex expressed on human T cells sharing determinants with B-type chronic lymphatic leukaemia. *J. Exp. Med.*, **151**, 1539.